# Multivalent MVA-vectored vaccine elicits EBV neutralizing antibodies in rhesus macaques that reduce EBV infection in humanized mice

**DOI:** 10.3389/fimmu.2024.1445209

**Published:** 2024-09-13

**Authors:** Gabriela M. Escalante, Ivana G. Reidel, Lorraine Z. Mutsvunguma, Simeon Cua, Brenda A. Tello, Esther Rodriguez, Mafalda A. Farelo, Cloe Zimmerman, Murali Muniraju, He Li, Aparna N. Govindan, Michael K. Axthelm, Scott W. Wong, Javier Gordon Ogembo

**Affiliations:** ^1^ Department of Immuno-Oncology, Beckman Research Institute of City of Hope, Duarte, CA, United States; ^2^ Irell & Manella Graduate School of Biological Sciences of City of Hope, Duarte, CA, United States; ^3^ Vaccine and Gene Therapy Institute, Oregon Health and Science University, Beaverton, OR, United States; ^4^ Oregon National Primate Research Center, Oregon Health and Science University, Beaverton, OR, United States; ^5^ Department of Molecular Microbiology and Immunology, Oregon Health and Science University, Portland, OR, United States

**Keywords:** Epstein-Barr virus, infectious mononucleosis, cancer, prophylactic vaccine, glycoprotein, neutralizing antibody, rhesus macaque, humanized mice

## Abstract

**Introduction:**

Epstein-Barr virus (EBV) is an oncogenic human herpesvirus associated with ~350,000 cases of lymphoid and epithelial malignancies every year, and is etiologically linked to infectious mononucleosis and multiple sclerosis. Despite four decades of research, no EBV vaccine candidate has yet reached licensure. Most previous vaccine attempts focused on a single viral entry glycoprotein, gp350, but recent data from clinical and pre-clinical studies, and the elucidation of viral entry mechanisms, support the inclusion of multiple entry glycoproteins in EBV vaccine design.

**Methods:**

Here we generated a modified vaccinia Ankara (MVA)-vectored EBV vaccine, MVA-EBV5-2, that targets five EBV entry glycoproteins, gp350, gB, and the gp42gHgL complex. We characterized the genetic and translational stability of the vaccine, followed by immunogenicity assessment in BALB/c mice and rhesus lymphocryptovirus-negative rhesus macaques as compared to a gp350-based MVA vaccine. Finally, we assessed the efficacy of MVA-EBV5-2-immune rhesus serum at preventing EBV infection in human CD34+ hematopoietic stem cell-reconstituted NSG mice, under two EBV challenge doses.

**Results:**

The MVA-EBV5-2 vaccine was genetically and translationally stable over 10 viral passages as shown by genetic and protein expression analysis, and when administered to female and male BALB/c mice, elicited serum EBV-specific IgG of both IgG1 and IgG2a subtypes with neutralizing activity *in vitro*. In Raji B cells, this neutralizing activity outperformed that of serum from mice immunized with a monovalent MVA-vectored gp350 vaccine. Similarly, MVA-EBV5-2 elicited EBV-specific IgG in rhesus macaques that were detected in both serum and saliva of immunized animals, with serum antibodies demonstrating neutralizing activity *in vitro* that outperformed serum from MVA-gp350-immunized macaques. Finally, pre-treatment with serum from MVA-EBV5-2-immunized macaques resulted in fewer EBV-infected mice in the two challenge experiments than pretreatment with serum from pre-immune macaques or macaques immunized with the monovalent gp350-based vaccine.

**Discussion:**

These results support the inclusion of multiple entry glycoproteins in EBV vaccine design and position our vaccine as a strong candidate for clinical translation.

## 1 Introduction

Epstein-Barr virus (EBV) is a gamma-herpesvirus prevalent in >90% of the human population (1). It was the first human oncogenic virus to be identified and is associated with approximately 350,000 new cases of epithelial and lymphoid malignancies every year (2). EBV is also the causative agent of infectious mononucleosis, is associated with several autoimmune diseases, and was recently established as a major causative factor in the development of multiple sclerosis (1, 3–5). Despite four decades of EBV vaccine research, no prophylactic vaccine against the virus or its associated diseases has yet been licensed (6).

Upon first contact with the oral mucosa, EBV utilizes five glycoproteins to achieve entry into its two main target cells, epithelial and B cells. These glycoproteins, gp350, gB, gp42, gH, and gL, are key targets of interest for developing an effective prophylactic vaccine (7). In epithelial cells, the virus attaches to ephrin receptor A2 on target cells via the heterodimeric complex gHgL (8, 9), which can also bind to non-muscle myosin heavy chain IIA (10). Binding of gHgL to its target receptors then activates the fusogenic activity of gB (7), which binds to neuropilin 1 on the target cell (11), culminating in viral entry (7). In B cells, EBV attaches to complement receptor type 1 (CR1/CD35) and/or type 2 (CR2/CD21) via gp350 (12, 13), triggering endocytosis of the virion (14). The fusion process is then carried out by gHgL in complex with gp42, which binds to MHC class II (15–17) and activates the fusogenic activity of gB. Of these five glycoproteins, gp350 dominated the field as the main immunogen tested during the first 20 years of EBV vaccine research (6). This culminated in four Phase I/II clinical trials that tested vaccines that targeted gp350 alone, but these were not successful in reducing EBV infection rates, and failed to move to Phase III clinical trials and achieve licensure (18–21). Knockout studies have shown that gp350 is not essential for viral entry (22). However, these same studies have shown that gp350 does serve to enhance infection, and all five glycoproteins are targets of neutralizing antibodies in both naturally infected individuals and in animal antibody and vaccine studies (6, 23–35). There are also reports that these glycoproteins can elicit cellular immune responses (36–44). Based on this evidence, we reasoned that a robust immune response against all five entry glycoproteins might be required for an EBV vaccine to achieve a sufficiently protective immune response against infection. Indeed, the field of EBV vaccine research has recently shifted toward multivalent vaccine approaches (6, 45, 46), and our group is focused on optimizing the inclusion of these glycoproteins in a single vaccine to stimulate robust immune responses to prevent primary EBV infection and its associated diseases (47, 48).

Previously, we developed a multivalent virus-like particle (VLP) that incorporated gp350, gB, and gp42gHgL as a prophylactic EBV vaccine (48). The vaccine was immunogenic in immunized rabbits, eliciting glycoprotein-specific IgG with higher neutralizing activity in epithelial and B cells than IgG elicited by a gp350-based vaccine, and on par with IgG elicited by immunization with UV-inactivated EBV (UV-EBV). Despite these successes, the VLP production process was not optimal for large-scale manufacturing. Thus, to improve immunogenicity and facilitate vaccine production, we adopted a viral vector, the modified vaccinia Ankara (MVA) virus, as a platform to express the five target glycoproteins. Of all available viable viral vaccine vectors, vaccinia vectors have the largest capacity to harbor foreign DNA (~25–30kb) (49, 50), and thus are the only vectors with enough genetic capacity to harbor the sequences of our five target EBV glycoproteins (~10 kb). MVA is highly immunogenic and was derived from the chorioallantois vaccinia virus Ankara strain after extensive passaging in culture (51, 52). Through this passaging, MVA lost its ability to productively replicate in human cells, rendering it an extremely safe vector (51, 53). The immunogenicity and safety of MVA has been confirmed in multiple vaccine clinical trials, including in immunosuppressed individuals, and multiple MVA-based vaccines are currently in the different stages of development pipeline against various pathogens, including Ebola, HIV, influenza, cytomegalovirus, and SARS-CoV-2, among others (52, 54–60).

Here we present the design, development, characterization, and immunogenicity of an MVA-vectored multivalent vaccine candidate that incorporates gp350, gB and the gp42gHgL complex. The vaccine was found to be stable over ten viral passages, maintaining expression of all five glycoproteins in infected cells. In immunized BALB/c mice, the vaccine elicited glycoprotein-specific IgG against the target glycoproteins, with neutralizing activity against EBV B cell infection *in vitro* that outperformed neutralizing activity elicited by immunization with UV-EBV or a monovalent MVA-gp350 vaccine. Importantly, we replicated these results in non-human primate (NHP) rhesus macaque studies, in which we were additionally able to detect glycoprotein-specific IgG in the saliva of immunized animals. To further assess the neutralizing activity elicited by the vaccine in rhesus macaques *in vivo*, we performed passive immunization experiments in humanized mice in two independent experiments testing distinct EBV challenge doses, in which the multivalent vaccine displayed a protective effect against EBV infection in mice. Overall, our results suggest that the multivalent vaccine provides better neutralization of B-cell infection compared to a monovalent vaccine. Together, these results support our multivalent vaccine approach and position our vaccine as a strong candidate for clinical translation.

## 2 Materials and methods

For material and reagent catalog numbers and additional information, please refer to **Supplementary Table S1**.

### 2.1 Cell lines, primary cells, and viruses

All cell lines were incubated at 37°C in the presence of 5% CO_2_ and grown in media supplemented with 10% FBS (Genesee Scientific), 1% L-glutamine (ThermoFisher Scientific), 2% penicillin-streptomycin (ThermoFisher Scientific) unless otherwise noted, and were tested for mycoplasma contamination. BHK-21 (ATCC CCL-10) are Syrian golden hamster kidney cells and were grown in DMEM (Corning) media. AGS-Akata-EBV-eGFP are human female gastric adenocarcinoma cells harboring EBV in which the thymidine kinase (TK) gene has been replaced with a neomycin and GFP cassette [Akata-EBV-eGFP, (27)] that were a kind gift from Dr. Lindsey Hutt-Fletcher and Dr. Rona Scott (Louisiana State University, Baton Rouge, LA, USA); they were grown in DMEM/F-12 (Corning) media additionally supplemented with 500 µg/ml G418 (ThermoFisher Scientific). HEK-293 (ATCC CRL-1573) are human female embryonic kidney cells and were grown in DMEM media. Raji (ATCC CCL-86) are human male Burkitt lymphoma cells and were grown in RPMI (Corning) media. CEF (AVS Bio 10100807) are chicken embryo fibroblasts and were grown in VP-SFM media (Gibco) supplemented with 10% FBS and 1% GlutaMAX (Gibco). P3X63Ag8.653 (ATCC CRL-1580) are mouse plasmacytoma cells and were grown in RPMI media supplemented with 10% FBS, 2% L-glutamine, 1% penicillin-streptomycin, 1% sodium pyruvate (Corning), 0.6% HEPES (Lonza), and 0.1% 2-mercaptoethanol (Invitrogen). Hybridoma cells resulting from P3X63Ag8.653 and mouse splenocyte fusion were cultured in DMEM containing HAT (Gibco).

Primary human stem cells were purchased from Advanced Bioscience Resources following federal and state regulatory guidelines, and immediately processed for mouse humanization as described in “Human lymphocyte engraftment of NSG mice.” Advanced Bioscience Resources is a nonprofit organization compliant with human subject protection requirements, with its own Institutional Review Board.

Akata-EBV-eGFP virus was produced from AGS-Akata-EBV-eGFP cells, as described in Method Details. Recombinant MVA virus expressing EBV gp350, gB, gp42, gH, and gL or gp350 alone was constructed via bacterial artificial chromosome (BAC) technology from MVA-BAC-TK, and reconstituted and expanded in BHK-21 cells as described in Method Details. MVA-BAC-TK harbors the MVA genome with the BAC pBeloBAC11 (GenBank: U51113) inserted at the position of the TK gene together with an eGFP expression cassette. MVA-BAC-TK in GS1783 bacteria was a kind gift from Dr. Don Diamond (Beckman Research Institute of City of Hope, Duarte CA, USA), and was developed from the MVA 1974/NIH clone 1, obtained under MTA from Dr. Bernard Moss (National Institutes of Allergy and Infectious Diseases, Bethesda, MD, USA). MVA-BAC-TK has been previously described (61), as has GS1783 bacteria (62).

### 2.2 Mice

Female and male BALB/c mice aged 8–10 weeks purchased from Charles River Laboratories (Strain# 028) were used for vaccine immunogenicity studies.

Female and male NOD.Cg-*Prkdc^scid^ Il2rg^tm1Wjl^*/SzJ (NSG) mice obtained from an ongoing NSG colony at the Beckman Research Institute of City of Hope Animal Resource Center, originally purchased from The Jackson Laboratory (Strain# 005557; RRID: IMSR_JAX:005557), were used for vaccine efficacy studies. Prior to vaccine efficacy studies, 3–5-week-old NSG mice were engrafted with CD34+ human hematopoietic stem cells to generate humanized mice as described in Method Details.

All mice were housed at BSL2 facilities in the Animal Resource Center of Beckman Research Institute of City of Hope, with free access to food and water in a 12:12 light:dark cycle. The animal facilities are accredited by the Association for Assessment and Accreditation of Laboratory Animal Care (AAALAC) and conform to the Institute for Laboratory Animal Research Guide for the Care and Use of Laboratory Animals. All mouse procedures were performed in accordance with approved Beckman Research Institute of City of Hope Institutional Biosafety Committee (IBC, #16001) and Institutional Animal Care and Use Committee (IACUC, #16003 and #22059) protocols.

### 2.3 Rhesus macaques

Female and male Indian-origin rhesus macaques (*Macaca mulatta*) aged 9–24 years (see **Supplementary Figure S5**) were leased from and housed at the Oregon National Primate Research Center (ONPRC) and used for vaccine immunogenicity studies. Animals were housed in specific-pathogen-free facilities and periodically tested for rhLCV infection. The ONPRC is an AAALAC- accredited research facility and conforms to National Institutes of Health guidelines on the ethical use of animals in research. All rhesus macaque procedures were performed in accordance with approved ONPRC IACUC (#IP00003900) and Beckman Research Institute of City of Hope IBC (#16001) and IACUC (#16003 and #22059) protocols. Additional serum samples from non-SPF rhesus macaques used as controls in rhLCV seroreactivity tests were obtained from the Southwest National Primate Research Center at the Texas Biomedical Research Institute.

### 2.4 Plasmids, recombinant DNA, and oligonucleotides

All pCAGGS plasmid cloning and synthesis were performed by Genewiz at Azenta Life Sciences, using a pCAGGS parental plasmid that has been previously described (63). The mH5-Kan-gp350-2A-gB-pCAGGS plasmid contains a bi-cistronic expression cassette that codes for full-length gp350 (GenBank: CAD53417.1) and gB (GenBank: CAD53463.1), interspersed by a 2A autocleavable peptide sequence. The 2A peptide and associated sequences have been previously described (48, 64). The mH5-Kan-gp42-2A-gL-2A-gH-pCCAGS plasmid contains a tri-cistronic expression cassette that codes for full-length gp42 (GenBank: GenBank: CAD53422.1), gL (GenBank: CAD53428.1), and gH (GenBank: CAD53450.1), interspersed by 2A autocleavable peptide sequences. The mH5-Kan-gp350-pCAGGS, mH5-Kan-gB-pCAGGS, mH5-Kan-gp42-pCAGGS, mH5-Kan-gH-pCAGGS, and mH5-Kan-gL-pCAGGS plasmids contain expression cassettes that code for each glycoprotein individually, respectively. Expression cassettes for all pCAGGS plasmids are under the control of the modified H5 (mH5) promoter (65), which is followed by a I-SceI restriction enzyme site and the Kanamycin resistance (Kan R) gene flanked by two 40 bp duplication sequences, preceding the glycoprotein genes.

EBV-gp42-His-Avi-PTT3, EBV-gH-His-Avi-PTT3, and EBV-gL-His-Avi-PTT3 plasmids coding for EBV gp42 (a.a. 33–223, GenBank: AFY97939.1), gH (a.a. 19–679, GenBank: AFY97969.1), and gL (a.a. 24–137, GenBank: AFY97944.1), respectively, have been previously described (28) and were kind gifts from Dr. Andrew McGuire (Fred Hutchinson Cancer Center, Seattle, WA, USA).

PTT3 plasmid cloning and synthesis for rhLCV-gp350-ecto-His-Avi-PTT3 were performed by Genewiz at Azenta Life Sciences, using a PTT3 parental plasmid (National Research Council of Canada) that has been previously described (66, 67). The rhLCV-gp350-ecto-His-Avi-PTT3 plasmid contains an expression cassette that codes for the rhLCV gp350 ectodomain (a.a. 1–739, NCBI Reference sequence: NC_006146.1, Gene ID: 2949805) fused to a 6xHis-Avidin tag.

Synthesis for all primers and BALF5 FAM-labeled probe and gBlock was performed by Integrated DNA Technologies.

### 2.5 Generation of recombinant MVA vectors

Recombinant MVA vectors were generated using standard homologous recombination techniques. To generate MVA-EBV5-1, the glycoprotein expression cassette in the mH5-Kan-gp350-2A-gB-2A-gp42-2A-gL-2A-gH-pCAGGS plasmid was amplified using site-specific primers for homologous recombination that add 50 bp duplications of the desired MVA insertion site at each amplicon end (*en passant* 69R/70L primer pair). One round of *en passant* mutagenesis was subsequently carried out in GS1783 bacteria harboring MVA-BAC-TK (62), which resulted in the insertion of the expression cassette coding for gp350-2A-gB-2A-gp42-2A-gL-2A-gH into the 69R/70L MVA genomic site, and removal of the Kan R gene. To generate MVA-EBV5-2, MVA-EBV-gp350-gB and MVA-EBV-gp42gLgH, the glycoprotein expression cassettes in mH5-Kan-gp350-2A-gB-pCAGGS and mH5-Kan-gp42-2A-gL-2A-gH-pCCAGS plasmids were amplified using site-specific primers for homologous recombination (*en passant* 69R/70L and 64L/65L primer pairs, respectively). Two rounds of *en passant* mutagenesis were subsequently carried out in GS1783 bacteria harboring MVA-BAC-TK as above, which resulted in the insertion of expression cassettes coding for gp350-2A-gB and gp42-2A-gL-2A-gH into the 69R/70L (G1L) and 64L/65L (IGR3) MVA genomic sites, respectively, and removal of the Kan R gene from each. To generate MVA-EBV5-5, the glycoprotein expression cassettes in the mH5-Kan-gp350-pCAGGS, mH5-Kan-gB-pCAGGS, mH5-Kan-gp42-pCAGGS, mH5-Kan-gH-pCAGGS, and mH5-Kan-gL-pCAGGS plasmids were amplified using site-specific primers for homologous recombination (*en passant* 69R/70L, 64L/65L, Del3, 44L/45L and 148R/149L primer pairs, respectively). Five rounds of *en passant* mutagenesis were subsequently carried out in GS1783 bacteria harboring MVA-BAC-TK as above, which resulted in the insertion of expression cassettes coding for gp350, gB, gp42, gH and gL into the 69R/70L (G1L), 64L/65L (IGR3), Del3, 44L/45L and 148R/149L MVA genomic sites, respectively, and removal of the Kan R gene from each. To generate MVA-gp350, the sequence for mH5-Kan together with the gp350 ectodomain (a.a. 1-864) was first amplified from the mH5-Kan-gp350-2A-gB-pCAGGS plasmid using a site-specific primer for homologous recombination on the 5’ end (*en passant* Del3 forward primer), and a primer that adds a 6xHis tag on the 3’ end (gp350 ectodomain 6x tag Reverse primer). Then, the amplicon was further amplified with site-specific primers for homologous recombination on both ends (*en passant* Del3 primer pair). One round of *en passant* mutagenesis in GS1783 bacteria harboring MVA-BAC-TK proceeded as above, which resulted in the insertion of the expression cassette coding for gp350-ectodomain-6xHis in the Del3 MVA genomic site, and removal of the Kan R gene.

All recombinant MVA vectors described above were reconstituted by transfecting the respective purified recombinant DNA generated in GS1783 bacteria into BHK-21 cells previously seeded in a 6-well plate (1 µg DNA:4 µg polyethyleneimine [Sigma Aldrich] per well). Four hours after transfection, fowlpox virus was added to each transfected well. The next day, media was replaced with growth media, and the cells subsequently monitored for eGFP expression, as the MVA-BAC-TK backbones contain an eGFP marker. Over the following weeks, cells were maintained and grown until achieving 90–100% eGFP expression (full infection) in 18–20 150-mm cell culture dishes. To isolate the viruses, cells were harvested using a cell scraper, then pelleted by centrifugation at 335xg, at 4°C for 20 min, and supernatants discarded. Subsequently, cell pellets were frozen and thawed three times, then sonicated for 4 min. The resulting pellets were resuspended in Opti-MEM (ThermoFisher Scientific) and centrifuged at 4300xg, at 4°C for 10 min. The resulting supernatant, containing the virus (passage 0 [p0]), was then aliquoted and stored at -80°C until use.

For animal experiments, viruses were grown and expanded in BHK-21 cells for large-scale production, and purified via ultracentrifugation through a sucrose cushion as described in (68, 69). The resulting virus stocks were titrated in BHK-21 cells as described in (70) and in “Virus titrations” below, using eGFP as a marker for infection.

### 2.6 Genetic and translational stability assessment of MVA-EBV5-2

To assess the genetic and translational stability of MVA-EBV5-2, serial viral passaging was performed in BHK-21 cells. p0 stock was used to infect five 150-mm dishes of BHK-21 cells. Once infection had spread and 90–100% of the cells expressed eGFP, cells were processed for virus isolation as described above, generating p1. The process was repeated up to p10, and additional infected cells were harvested at each passage for DNA isolation and immunoblot analysis.

To assess the genetic stability of the inserted expression cassette, DNA from infected cells at each passage was isolated using the DNeasy Blood & Tissue Kit (QIAGEN) according to the manufacturer’s instructions. PCR was first used to assess the size of the two inserted glycoprotein expression cassettes by amplifying the sequences at the 69R/70L and 64L/65L MVA genomic sites. The resulting PCR products were analyzed using gel electrophoresis on an ethidium bromide 1% agarose gel. Additionally, p0 and p10 DNA was prepared and submitted to the City of Hope Integrative Genomics Core for full genome PacBio sequencing, according to the Core’s protocol.

To assess the translational stability of the virus, the isolated viruses were used to infect BHK-21 cell and assess expression of each individual glycoprotein via flow cytometry analysis, as described in “Flow cytometry-based assessment of glycoprotein expression in MVA vector-infected cells”. Additionally, total protein was isolated from the collected infected cells and processed for immunoblot as described in “Immunoblot-based assessment of glycoprotein expression in MVA vector-infected cells” to detect EBV gp350 and gp42.

### 2.7 Recombinant glycoproteins and antibodies

Recombinant EBV gp350/220 ectodomain (a.a.4–863) protein is available commercially (Immune Tech Corp.). Recombinant EBV gB (a.a. 23–683, GenBank: AFY97983.1) protein has been previously described (28) and was a kind gift from Dr. Andrew McGuire. Recombinant gp42gHgL (gH: a.a. 1–679, UniProtKB/Swiss-Prot: Q3KSQ3.1; gL: a.a. 1–137, UniProtKB/Swiss-Prot: P03212.1; gp42: a.a. 34–223, UniProtKB/Swiss-Prot: P0C6Z5.1) has been previously described (29) and was a kind gift from Dr. Jeffrey Cohen (National Institutes of Health, Bethesda, MD). Additional recombinant gp42gHgL (gp42: a.a. 33–223, GenBank: AFY97939.1; gH: a.a. 19–679, GenBank: AFY97969.1; gL: a.a. 24–137, Genbank: AFY97944.1) proteins were produced and purified by GenScript, via co-transfection of EBV-gp42-His-Avi-PTT3, EBV-gH-His-Avi-PTT3, and EBV-gL-His-Avi-PTT3 plasmids, respectively, in ExpiCHO-S cells using proprietary methods. Similarly, recombinant rhLCV gp350 ectodomain (a.a. 1–739, NCBI sequence: NC_006146.1, Gene ID: 2949805) protein was produced and purified by GenScript, via transfection of rhLCV-gp350-ecto-His-Avi-PTT3 plasmid in ExpoCHO-S cells. The purity of GenScript-produced proteins was assessed by the company via SDS-PAGE analysis, and further verified upon receipt in our laboratory via additional SDS-PAGE and immunoblot analyses, using anti-His primary antibody or glycoprotein-specific antibodies when available for immunoblot. Recombinant gp42 (a.a. 33–223, GenBank: AFY97939.1) used in 15C8 antibody characterization has been previously described (28) and was a kind gift from Dr. Andrew McGuire.

The murine antibodies F-2-1, CL40, and E1D1 against EBV gp42, gH, and gL, respectively, have been previously described (25–27) and were kind gifts from Dr. Lindsey Hutt-Fletcher and Dr. Rona Scott. The murine antibody HB5 against EBV gp350 has been previously described (30), and was produced in-house at the Ogembo laboratory as described (30) and purified at the X-Ray Crystallography and Macromolecular Characterization Core at the Beckman Research Institute of City of Hope. The human antibody AMMO5 against gB has been previously described (28) and was a kind gift from Dr. Andew McGuire. The murine antibody 72A1 against gp350 has been previously described (23) and was produced and purified by GenScript based on published sequences (30). The human antibody AMMO1 against gHgL has been previously described (28) and was produced and purified by GenScript based on published sequences (GenBank Accession Numbers KY631780.1 [VL] and KY631779.1 [VH]). Rat hybridoma 19C2 against vaccinia B5R has been previously described (71), and supernatant originating from this hybridoma was a kind gift from Dr. Don Diamond. The murine antibody 15C8 against EBV gp42 was generated using the traditional mouse hybridoma method as described for HB5 (30), and produced and purified as above for HB5. In brief, splenocytes from BALB/c mice immunized with gp42gHgL immunogens were fused with P3X63Ag8.653 cells at a 1:1 ratio using polyethylene glycol (Sigma)-mediated chemical fusion to generate hybridomas; supernatants from successfully grown hybridomas in 96-well plates were screened by ELISA using recombinant gp42 as the capture antigen, which yielded 101 positive hybridomas. Subsequently, these hybridomas were expanded to 48-well plates, and the resulting supernatants were screened again by ELISA for gp42 binding, yielding 11 positive hybridomas. These clones were further expanded until 100 ml of supernatant was collected for purification, which was tested a third time by ELISA; 9 clones remained positive, and supernatant IgG antibodies from 8 of the clones were purified using protein G affinity chromatography at the City of Hope X-Ray Crystallography Core. After purification, 4/8 antibodies remained positive by ELISA, and were subsequently further tested for gp42 binding by immunoblot, which yielded 2 positive clones, including 15C8. The complementary-determining regions of 15C8 were sequenced by GenScript, and cloned into an antibody expression plasmid, which was then used to produce and purify recombinant 15C8 antibody, the specificity of which was then re-confirmed by immunoblot.

### 2.8 Hybridoma screening by ELISA and immunoblot

Unpurified and purified supernatants from hybridoma culture were sequentially tested for gp42-specific antibody production during expansion using soluble recombinant gp42 as target antigen. Ninety-six-well Costar flat-bottom microplates (Corning Incorporated) were coated overnight at 4°C with 25 ng/well of recombinant gp42 in PBS (50 µl at 0.5 µg/ml). Plates were blocked with 100 µl BSA blocking buffer (3% BSA in 0.1% Tween-20 PBS) for 1 hour at room temperature, shaking. One hundred µl of unpurified hybridoma supernatant or 50 µl of 50 µg/ml purified hybridoma supernatant IgG was added to each well, in triplicate, except for the first unpurified supernatant ELISA screen, which was performed in singlets. Plates were incubated for 2 hours at room temperature, shaking, followed by incubation for 1 hour at room temperature, shaking, with 50 µl of HRP-conjugated anti-mouse IgG (1/2000 dilution in PBS). Between each step, plates were washed three times with 300 µl wash buffer (0.1% Tween-20 PBS). Plates were then incubated for 20 min with 100 µl of ABTS 2-Component Microwell Peroxidase Substrate (LGC SeraCare). Reactions were stopped using 100 µl of ABTS Peroxidase Stop Solution (LGC SeraCare), and the OD of the reactions was read at 405 nm with a Filter Max F3 microplate reader (Molecular Devices).

Purified hybridoma supernatant IgG samples that tested positive for gp42 binding by ELISA were further tested by immunoblot, as described below for rhLCV-gp350 in “Immunoblot-based assessment of glycoprotein expression in MVA vector-infected cells and purified rhLCV-gp350,” using recombinant gp42 as the target antigen, and a primary antibody concentration of 10 µg/ml. Purified recombinant 15C8 IgG after molecular cloning was further tested in immunoblot using purified recombinant gp42 as the target antigen, or lysates of uninfected BHK-21 cells, BHK-21 cells infected with empty MVA virus, or BHK-21 cells infected with MVA-EBV5-2 virus (p1), which are described below in “Immunoblot-based assessment of glycoprotein expression in MVA vector-infected cells and purified rhLCV-gp350.” Recombinant gp42 or cell lysates were prepared for SDS-PAGE by boiling with an appropriate volume of 6x reducing Laemmli SDS sample buffer (ThermoFisher Scientific) at 100°C for 5 min, and loading the samples on a Bolt 4-12%, Bis-Tris Plus gel (ThermoFisher Scientific). After gel electrophoresis, proteins were transferred to a nitrocellulose membrane via the iBlot 2 system (ThermoFisher Scientific), and subsequently incubated in 5% nonfat milk in 0.1% Tween-20 PBS for 1 hour at room temperature, shaking. The membranes were then incubated overnight at 4°C, shaking, with primary antibody diluted in 1% nonfat milk in 0.1% Tween-20 PBS, 10 µg/ml for 15C8 antibody, a 1/1000 dilution for mouse anti-His antibody (Invitrogen) and a 1/10 dilution for 19C2 rat hybridoma supernatant. Membranes were washed three times with wash buffer for 5 min, shaking. They were then incubated for 1 hour at room temperature, shaking, with secondary antibody diluted in 1% milk in 0.1% Tween-20 PBS, 1/2500 for both HRP-conjugated anti-mouse and anti-rat IgG. The membranes were then washed as before and briefly incubated with SuperSignal West Pico PLUS Chemiluminescent Substrate (ThermoFisher Scientific), after which they were immediately imaged in a PXi Multi-Application Gel Imaging System (Syngene). For Coomassie stain analyses, protein gels after electrophoresis were placed in Coomassie brilliant blue staining solution (Bio-Rad) for 12 hours, shaking, and subsequently destained by incubation in destaining solution (10% acetic acid, 40% methanol in deionized water) for 12 hours, shaking; resulting gels were imaged in a PXi Multi-Application Gel Imaging System.

### 2.9 Immunoblot-based assessment of glycoprotein expression in MVA vector-infected cells and purified rhLCV-gp350

Uninfected BHK-21 cells, BHK-21 cells that had been infected overnight with empty MVA or MVA-EBV5-2 viruses, or infected cells obtained from serial passaging of MVA-EBV5-2 were harvested using a cell scraper, then pelleted using centrifugation at 335xg at 4°C, for 10 min. In an additional experiment, BHK-21 cells were infected overnight with MVA-gp350 virus, and similarly harvested. Pellets were washed twice with cold PBS, then lysed with Mammalian Cell Lysis Buffer (GoldBio) treated with Pierce Protease Inhibitor (ThermoFisher Scientific). The resulting cell lysates, or recombinant rhLCV-gp350, were prepared for SDS-PAGE by boiling with an appropriate volume of 6x reducing Laemmli SDS sample buffer at 100°C for 5 min, and loading the samples on a Bolt 4-12%, Bis-Tris Plus gel. After gel electrophoresis, proteins were transferred to a nitrocellulose membrane via the iBlot 2 system, and subsequently incubated in BSA blocking buffer for 1 hour at room temperature, shaking. The membranes were then incubated overnight at 4°C, shaking, with primary antibody diluted in blocking buffer, 10 µg/ml for purified HB5 or 15C8 antibodies, a 1/1000 dilution for mouse anti-His antibody, a 1/100 dilution for polyclonal rhesus serum (rhLCV-positive animal from the study [ID 33466], Pre-immune serum), and a 1/10 dilution for 19C2 rat hybridoma supernatant. Membranes were washed three times with wash buffer for 5 min, shaking. They were then incubated for 1 hour at room temperature, shaking, with secondary antibody diluted in BSA blocking buffer, 1/2000 for HRP-conjugated anti-mouse and anti-rhesus IgG, and 1/2500 for HRP-conjugated anti-rat IgG. The membranes were then washed as before and briefly incubated with SuperSignal West Pico PLUS Chemiluminescent Substrate, after which they were immediately imaged in a PXi Multi-Application Gel Imaging System. Coomassie stain analyses for rhLCV-gp350 protein were performed similarly to analyses described above for gp42.

### 2.10 Flow cytometry-based assessment of glycoprotein expression in MVA vector-infected cells

BHK-21 cells previously seeded in a 48-well plate were infected with the different MVA viruses in triplicate per each glycoprotein/antibody to be tested and incubated overnight. Cells from each well were harvested and washed with PBS twice, after which they were incubated for 1 hour at room temperature with glycoprotein-specific primary antibody diluted in PBS (5 µg/ml). Cells were washed with PBS twice and were then incubated for 1 hour at room temperature with corresponding Alexa Fluor-647-conjugated secondary antibody diluted in PBS (1/2000). Cells were washed twice with PBS, and then fixed in 1% paraformaldehyde (PFA, Electron Microscopy Sciences). Stained cells were subsequently analyzed via flow cytometry in a BD Accuri C6 Flow Cytometer (BD). The obtained data was analyzed using FlowJo software and results were plotted using GraphPad Prism software. Plotted bar graphs depict Alexa Fluor-647-stained cell quantification following single cell and eGFP-positive cell (MVA-infected) gating (i.e. Alexa-Fluor-647-stained cells within the eGFP-positive single cell population).

### 2.11 Virus titrations

For Akata-EBV-eGFP titrations, flow cytometry based-titration was performed. Raji cells or HEK-293 cells previously seeded in 96-well plates or 48-well plates, respectively, were infected with various volumes (0-50 µl) of Akata-EBV-eGFP in triplicate and incubated overnight. Cells from each well were harvested and washed with PBS twice, after which they were fixed in 1% PFA. Cells were subsequently analyzed for eGFP expression via flow cytometry in a BD Accuri C6 Flow Cytometer or NovoCyte Quanteon 4025 (Agilent). The obtained data was analyzed using FlowJo software and results were plotted using GraphPad Prism software. Raji infectious units (Raji IU) per volume were calculated for Raji titrations by using the following formula, described in (72): Raji IU/volume of virus = (number of cells at time of infection × percent of eGFP-positive cells)/volume of inoculum. The formula was applied to each datapoint in the linear range of each infection curve obtained, and values averaged to obtain Raji IU/volume.

For titrations of MVA viruses used in immunization protocols, titrations were performed in BHK-21 cells as described above for Akata-EBV-eGFP titrations in HEK-293 cells. Flow cytometry analysis of eGFP expression was performed using a BD Accuri C6 Flow Cytometer and the obtained data was analyzed using FlowJo software. To calculate relative IU (RIU) per volume, the following formula was applied, as described in (70): for >30% eGFP-positive cells, RIU/volume of virus = cell number at the time of infection × [-LN(1-[p/100])] × (viral dilution factor/volume of inoculum); for <30% eGFP-positive cells, RIU/volume of virus = cell number at the time of infection × (p/100) × (viral dilution factor/volume of inoculum). The formula was applied to a single datapoint in the linear range of the infection curve obtained to calculate the RIU/volume.

To determine MVA-EBV5-2 titer stability after 1 or 5 months of storage at -80°C, a plaque-forming unit (PFU) immunostaining assay was performed as previously described (68, 73), with some modifications. Cells were seeded in 6-well plate at a density of 1,000,000 cells/well and incubated overnight. The next day, media was removed, and cells infected with 5 ten-fold serial dilutions (1/10^5^ to 1/10^9^) of virus in FBS-free growth media by adding 1 ml/well of inoculum in duplicate, followed by incubation for 2 hours at 37°C. Subsequently, 4 ml of complete growth media were added to each well, and the cells were incubated overnight. The next day, media was removed from wells and cells were washed with PBS before fixation in acetone:methanol (1:1). Cells were washed and stored in PBS at 4°C until immunostaining was performed. Staining was performed using the VECTASTAIN^®^ ABC-HRP Peroxidase (Rabbit IgG) and Peroxidase (HRP) DAB Substrate kits (Vector Laboratories) according to the manufacturer’s instructions with primary anti-vaccinia polyclonal antibody (Bio-Rad) diluted 1/2000 in PBS. Plaque identification and counting was done using an EVOS™ FL Digital Inverted Fluorescence Microscope (Invitrogen). Infectious units per ml (IU/ml) were calculated by multiplying the average plaque count between duplicates by the dilution factor and dividing by the inoculum volume. Only positive well counts between 10 - 100 plaques were selected for measurement.

### 2.12 EBV-reporter virus production

To produce GFP reporter virus (Akata-EBV-eGFP), AGS-Akata-EBV-eGFP cells were cultured in 15-cm dishes until reaching ~90% confluency. Growth media was replaced with DMEM/F-12 containing 10% FBS, 2% pen-strep, 33 ng/ml 12-O-tetradecanoylphorbol-13-acetate, and 2 mM sodium butyrate to induce viral lytic replication, and cells were subsequently incubated for 24 hours. After the incubation, the induction media was replaced with growth media without G418, and cells were incubated for an additional 5-7 days. Cell supernatants were collected and centrifuged at 4300xg for 90 min, at 4°°C. The resulting supernatants were filtered to remove cell debris (0.8 µm), followed by two sequential ultra-centrifugations in Beckman-Coulter type 19 rotors at 38,200xg for 90 min at 4°C to concentrate the virus. The resulting pellets at each centrifugation were resuspended in Opti-MEM, pooled, and stored at -80°C until use.

### 2.13 Immunizations in BALB/c mice

To assess the immunogenicity of MVA-EBV5-5, 8–10-week-old female BALB/c mice were immunized intraperitoneally on Day 0 and Day 28 for a total of two times with 5x10^7^ RIU of MVA-EBV5-5 (n=8/group). Blood was collected retro-orbitally 7 days prior to primary immunization, and on Day 49. For the main MVA-EBV5-2 immunogenicity experiment, 8–10-week-old female BALB/c mice were immunized intraperitoneally on Day 0, Day 28, and Day 56 for a total of three times with 5x10^7^ RIU of MVA-EBV5-2, MVA-gp350, or empty MVA, or with 5x10^4^ Raji IU of UV-inactivated EBV (UV-EBV) (n=8/group). Blood was collected from the submandibular vein 7 days prior to primary immunization, and on Days 21, 49, 84, and 119, after which the animals were terminally bled on Day 182 via cardiac puncture. The immunization was then repeated identically in 8–10-week-old male BALB/c mice, with the exception that mice were terminally bled on Day 119. *For the welfare of the animals, we made the decision to terminate the male mouse experiment earlier due to increasing fighting among cage mates that was leading to severe injuries that necessitated euthanasia.* In all cases, serum was separated from whole blood by collecting blood in serum collection tubes containing a clotting activator and centrifuging collection tubes at 10,000xg for 5 min.

### 2.14 Measurement of rhLCV seroreactivity in rhesus macaques by ELISA

Serum anti-rhLCV-gp350 IgG in rhesus macaques prior to immunization was measured via ELISA using soluble recombinant rhLCV gp350 as the target antigen. Ninety-six-well Nunc MaxiSorp flat-bottom microplates (ThermoFisher Scientific) were coated overnight at 4°C with 100 ng/well of the target antigen in PBS (100 µl at 1 µg/ml). Plates were blocked with 200 µl blocking buffer for 1 hour at room temperature, shaking. One hundred µl of serum from individual animals diluted 1/100 in PBS was then added to the plates in duplicate. Plates were incubated for 2 hours at room temperature, shaking, followed by incubation for 1 hour at room temperature, shaking, with 100 µl of HRP-conjugated anti-rhesus IgG diluted 1/5000 in PBS. Between each step, plates were washed three times with 300 µl wash buffer. Lastly, plates were incubated for 20 min with 100 µl of ABTS 2-Component Microwell Peroxidase Substrate. Reactions were stopped with 100 µl of ABTS Peroxidase Stop Solution, and the optical density (OD) of the reactions was read at 405 nm with a Filter Max F3 microplate reader. The obtained data was analyzed and plotted using GraphPad Prism software.

### 2.15 Immunizations in rhesus macaques

Nine to twenty-four-year-old female and male Indian-origin rhesus macaques (see **Supplementary Figure S5**) were immunized intramuscularly on Day 0 and Day 28 for a total of two times with 1x10^8^ RIU of MVA-EBV5-2 or MVA-gp350, or with 1x10^5^ Raji IU of UV-EBV (n=5/group). Blood and saliva were collected under ketamine sedation 7 days prior to primary immunization, and on Days 21, 56, 84, 112, 140, 168, and 196. Blood was collected using venipuncture via the saphenous vein; serum was separated from whole blood by collecting blood in serum collection tubes containing a clotting activator and centrifuging collection tubes at 1000xg for 10 min. Saliva was collected using saliva collection swabs by placing the swab in both the left and right cheeks of the rhesus macaques. The swabs were left in the mouth for 1–3 min to allow production and absorption of saliva, after which they were placed back in the swab container, and 1 mL PBS added. Swabs were subsequently spun down in their containers at 1000xg for 10 min.

### 2.16 Measurement of glycoprotein-specific antibodies by ELISA

Glycoprotein-specific IgG in immunized mice (total IgG, IgG1, or IgG2a) was measured via ELISA using soluble recombinant gp350, gB, and gp42gHgL as target antigens. Ninety-six-well Nunc MaxiSorp flat-bottom microplates were coated overnight at 4°C with 25 ng/well of the target antigen in PBS (50 µl at 0.5 µg/ml). Plates were blocked with either 100 µl milk blocking buffer (5% nonfat milk in PBS, total IgG) or BSA blocking buffer (IgG1 and IgG2a) for 1 hour at room temperature, shaking. Equal amounts of serum from each animal for each treatment group and timepoint were pooled, diluted 1/900 in 1% nonfat milk in PBS (total IgG), and added to the plates in triplicate (50 µl). Alternatively, individual mouse serum samples were assessed at a 1/900 dilution in PBS (IgG1 and IgG2a). Plates were incubated for 2 hours at room temperature, shaking, followed by incubation for 1 hour at room temperature, shaking, with 50 µl of either HRP-conjugated anti-mouse IgG (1/2000 dilution in 1% nonfat milk in PBS), IgG1 (1/2000 dilution in PBS), or IgG2a (1/10,000 dilution in PBS). Between each step, plates were washed three times with 300 µl wash buffer. Lastly, plates were incubated for 20 min with 100 µl of ABTS 2-Component Microwell Peroxidase Substrate (LGC SeraCare, Milford, MA, USA). Reactions were stopped using 100 µl of ABTS Peroxidase Stop Solution (LGC SeraCare), and the OD of the reactions was read at 405 nm with a Filter Max F3 microplate reader (Molecular Devices). The obtained data was analyzed and plotted using GraphPad Prism software.

Serum and saliva glycoprotein-specific IgG and saliva glycoprotein-specific IgA of immunized rhesus macaques were measured via ELISA using soluble recombinant gp350, gB, and gp42gHgL as target antigens. *Note that saliva assays were performed using BSA as a blocking agent and the saliva samples exhausted before we optimized the use of milk blocking*. Ninety-six-well Nunc MaxiSorp flat-bottom microplates were coated overnight at 4°C with 50 ng/well of the target antigen in PBS (100 µl at 0.5 µg/ml for serum assays, 50 µl at 1 µg/ml for saliva assays). Plates were blocked with either 200 µl of milk blocking buffer or 100 µl of BSA blocking buffer for serum or saliva assays, respectively, 1 hour at room temperature, shaking. One hundred µl of serum from individual animals diluted 1/100 in 1% nonfat milk in PBS, or 50 µl of undiluted saliva from individual animals were then added to the plates in duplicate. Plates were incubated for 2 hours at room temperature, shaking, followed by incubation for 1 hour at room temperature, shaking, with either 100 µl of HRP-conjugated anti-rhesus IgG diluted 1/5000 in 1% nonfat milk in PBS for serum assays, or 50 µl HRP-conjugated anti-rhesus IgG diluted 1/1000 in PBS for saliva assays. For measurement of saliva IgA, after saliva incubation, plates were incubated for 2 hours at room temperature with 50 µl anti-rhesus IgA diluted 1/500 in PBS, shaking, followed by incubation with HRP-conjugated anti-mouse IgG diluted 1/1000 in PBS for 1 hour at room temperature, shaking. Between each step, plates were washed three times with 300µl wash buffer. Lastly, plates were incubated for 20 min with 100 µl of ABTS 2-Component Microwell Peroxidase Substrate. Reactions were stopped with 100 µl of ABTS Peroxidase Stop Solution, and the OD of the reactions was read at 405 nm using a Filter Max F3 microplate reader. The obtained data was analyzed and plotted using GraphPad Prism software.

### 2.17 MVA infectivity assessment in epithelial and B cells

For MVA and MVA-EBV5-2 infectivity assessment in epithelial (HEK-293) and B (Raji) cells, cell seeding, infection, and processing were performed as described below in “Epithelial cell neutralization assay” and “B cell neutralization assay,” with a few changes. Cells were infected with MVA or MVA-EBV5-2 viruses at an MOI of 0.5, or as a control, cells were infected with Akata-EBV-eGFP virus, at a volume expected to yield ~14% infection. For neutralization assessment using EBV-specific 72A1 and AMMO1 neutralizing antibodies, viruses were incubated with 25 µg/ml of either antibody in Opti-MEM.

### 2.18 Epithelial cell neutralization assay

For epithelial cell neutralization assays using mouse sera, HEK-293 cells were seeded in a 48-well plate at a density of 50,000 cells/well and incubated overnight. The next day, Akata-EBV-eGFP virus, at a volume expected to yield 10–15% infection (real yield ~13% for female assay, ~17% for male assay), was incubated with serially diluted pooled immune mouse sera in a total of 100 µl of Opti-MEM per well for 1 hour at 37°C. Following the incubation, the virus/sera mixture was added to triplicate wells and incubated for another hour at 37°C. Infection media was removed and 300 µl of growth media was subsequently added to each well, and the cells were incubated overnight. The next day, cells were processed for flow cytometry as described above for Akata-EBV-eGFP titration in HEK-293 cells. Percent neutralization values for every dilution were calculated by using the following formula: percent neutralization = 100-(% eGFP-positive cells × 100/% eGFP-positive cells in negative control sample), for which serum-free infection was used as the negative control reference sample.

For epithelial cell neutralization assays using rhesus macaque sera, HEK-293 cells were seeded in a 48-well plate at a density of 75,000 cells/well and incubated overnight. The next day, Akata-EBV-eGFP virus, at a volume expected to yield 10–15% infection (real yield ~25% infection), was incubated with serially diluted individual rhesus macaque serum in a total of 100 µl of Opti-MEM per well for 1 hour at 37°C. Following the incubation, the virus/serum mixture was added to duplicate wells and incubated for another hour at 37°C. Five hundred µl of growth media was subsequently added to each well, and the cells were incubated overnight. The next day, cells were processed for flow cytometry as described above for Akata-EBV-eGFP titration in HEK-293 cells. Percent neutralization for every animal at each dilution was calculated using the formula described above, using Pre-immune serum from each corresponding animal as the negative control reference sample. The IC50 and IC80 for each sample were calculated as the last serum dilution in which ≥50% and ≥80% neutralization were achieved, respectively.

### 2.19 B cell neutralization assay

For B cell neutralization assays using mouse sera, Raji cells were seeded in a 96-well plate at a density of 50,000 cells/well in 50 µl of Opti-MEM. The same day, Akata-EBV-eGFP virus, at a volume expected to yield 10–15% infection (real yield ~5% infection), was incubated with serially diluted pooled immune mouse sera in a total of 50 µl of Opti-MEM per well for 1 hour at 37°C. Following the incubation, the virus/serum mixture was added to duplicate wells and incubated for another hour at 37°C. Two hundred-fifty µl of growth media was subsequently added to each well, and the cells were incubated overnight. The next day, cells were processed for flow cytometry as described above for Akata-EBV-eGFP titration in Raji cells. The percent neutralization for each dilution was calculated using the formula described above for HEK-293 cells.

For B cell neutralization assays using rhesus macaque sera, Raji cells were seeded in a 96-well plate at a density of 50,000 cells/well in 50 µl of Opti-MEM. The same day, Akata-EBV-eGFP virus, at a volume expected to yield 10–15% infection (real yield ~5% infection), was incubated with serially diluted individual rhesus macaque serum in a total of 50 µl of Opti-MEM per well for 1 hour at 37°C. Following the incubation, the virus/serum mixture was added to duplicate wells and incubated for another hour 37°C. Two hundred-fifty µl of growth media was subsequently added to each well, and the cells were incubated overnight. The next day, cells were processed for flow cytometry as described above for Akata-EBV-eGFP titration in Raji cells. The percent neutralization for every animal at each dilution was calculated using the formula described above for HEK-293 cells, using Pre-immune serum from each corresponding animal as the negative control reference sample. IC50 and IC80 for each sample were calculated as the last serum dilution at which ≥50% and ≥80% neutralization were achieved, respectively.

### 2.20 Depletion of glycoprotein-specific antibodies from immune rhesus macaque sera

To deplete gp350-specific antibodies from rhesus macaque sera, equal volumes of Day 56 immune macaque serum from every animal were pooled per treatment group. Nitrocellulose membranes (Thermo Scientific) cut into 0.5 x 3 cm strips were first incubated for 2 hours in 2 mL tubes with 500 µl of either PBS or PBS containing 25 µg of gp350 protein, shaking. Membranes were subsequently washed three times with 0.05% Tween-20 PBS, and then incubated with 2 mL of BSA blocking buffer for 1 hour, shaking. Membranes were washed as before, then incubated overnight at 4°C with 500 µl of pooled rhesus macaque sera diluted 1/12.5 in PBS. To verify anti-gp350 depletion in the pooled sera, ELISAs were performed similarly as described in “Measurement of glycoprotein-specific antibodies by ELISA” for individual animal sera, using BSA blocking buffer and a pooled sera dilution of 1/50 in PBS, with secondary antibody diluted in PBS. The sera were subsequently evaluated in neutralization assays as described in “Epithelial cell neutralization assay” and “B cell neutralization assay” above, using undepleted sera as a control, and using virus volumes expected to yield 10–15% infection in epithelial and B cell assays (real yield ~16% HEK-293 infection and ~4% Raji infection). To further study the contribution of antibodies against the different target glycoproteins to neutralization in MVA-EBV5-2 sera, we repeated this process using only serum from MVA-EBV5-2 macaques, and depleting antibodies against gp350, gB and gp42gHgL. In this case real infection yields were ~12% in HEK-293 and ~30% in Raji.

### 2.21 Human lymphocyte engraftment of NSG mice

To humanize NSG mice, 3–5-week-old NSG mice were engrafted with CD34+ human hematopoietic stem cells. Upon receipt from the vendor, human stem cells were enriched for CD34+ hematopoietic stem cells using an EasySep Human CD34 Positive Selection Kit II (STEMCELL) per the manufacturer’s instructions. Mice were then subjected to 240 cGY of whole-body radiation, followed by a retro-orbital injection of 1x10^5^ CD34-enriched human cells per mouse. Twelve weeks post-engraftment, humanization in each mouse was assessed via flow cytometry. Blood was collected retro-orbitally from each mouse in EDTA-coated tubes, and subsequently incubated with 2 µl PE-conjugated anti-human CD45 antibody at 4°C for 30 min in the dark. Two ml red blood cell lysis buffer (Sigma Aldrich) was then added to lyse red blood cells and samples were incubated for 10 min in the dark at room temperature. Cell suspensions were centrifuged at 450xg for 5 min, supernatants were removed, and cells were washed with 200 µl of PBS. Cell suspensions were then centrifuged at 600xg for 5 min, supernatants were removed, and cells were fixed in 2% PFA. Stained cells were analyzed via flow cytometry using an LSRFortessa Cell Analyzer (BD) to determine the percentage of human CD45 (hCD45) lymphocytes in every animal. The obtained data was analyzed using FlowJo software and results were plotted using GraphPad Prism software. NSG mice with successful human lymphocyte engraftment (humanized) are herein referred to as NSG huMice.

### 2.22 Rhesus macaque serum kinetics in NSG huMice

NSG huMice mice (n=12) were immunized intraperitoneally with 500 µl of pooled Day 56 rhesus macaque UV-EBV-immune sera. Mice were then sequentially terminally bled using cardiac puncture at 3, 6, 12, and 24 hours post-immunization (n=3/timepoint); n=3 untreated mice were also terminally bled at the start of the experiment. The levels of gp350-specific rhesus IgG in the sera of NSG huMice were determined by ELISA at each timepoint, similarly to described above for sera of immunized rhesus macaques, with a few differences: plates were coated with 100 ng/well of target antigen (100 µl at 1 µg/ml) and blocked with BSA blocking buffer, mouse sera was diluted 1/50 in PBS, and secondary antibody was diluted in PBS. In addition, the intraperitoneal cavity of each mouse was inspected after euthanasia for the presence of undiffused rhesus macaque sera at each timepoint post-immunization.

### 2.23 EBV challenge in NSG huMice

Two independent challenge experiments were performed. Prior to start of the studies, NSG huMice were sorted and randomly allocated into treatment groups using a balanced allocation algorithm according to the % hCD45 lymphocytes in each animal via WINPEPI software (74), to ensure similar distribution of humanization levels across treatment groups. In both experiments, mice were immunized intraperitoneally with 500 µl of pooled rhesus macaque sera from either Day -7 (all animals, Pre-immune), Day 56 MVA-EBV5-2 animals, or Day 56 MVA-gp350 animals (n=7/group). Twelve hours post-immunization, NSG huMice were challenged intraperitoneally with Akata-EBV-eGFP. In the low-dose experiment, mice received 5x10^3^ Raji IU Akata-EBV-eGFP and were monitored for 56 days; n=2 additional mice were neither immunized nor challenged to serve as a Sham control. In the high-dose study, mice received 5x10^4^ Raji IU Akata-EBV-eGFP and were monitored for 28 days; n=6 additional mice were neither immunized nor challenged to serve as a Sham control. After the observation period, mice were terminally bled, and their spleens were collected. Spleens were imaged using a standard smartphone camera, and the perimeter and area of each spleen were quantified from these images using IC Measure (The Imaging Source) software. Spleens were divided in half, and each half was processed differently to assess infection outcomes. One half was processed for immunohistochemistry and EBV-encoded small RNA (EBER) *in situ* hybridization (ish). Spleens were fixed in 10% formaldehyde, then stored in 70% ethanol before submission for processing to the City of Hope Pathology Solid Tumor Core. The other half was processed along with blood for infection detection via qPCR as described in “Quantitative PCR analysis of human cells in humanized mice.”

### 2.24 Quantitative PCR analysis of human cells in humanized mice

To assess viremia and infection outcomes in EBV-challenged humanized mice, DNA was first isolated from collected blood and spleens using the DNeasy Blood & Tissue Kit according to the manufacturer’s instructions. For blood, 70 µl were processed per animal, and the resulting DNA was eluted in 50 µl of elution buffer. TaqMan qPCR reactions were then prepared using EBV BALF5-specific primers and probe. Each 25 µl reaction contained 1.25 µl of primer-probe mix (600 nM of each primer, 300 nM of FAM-labeled probe), 1.25 µl of TaqMan 20x VIC-labeled human RNase-P primer-probe mix, 12.5 µl of 2x QuantiTect Probe PCR Master Mix, and 10 µl of test sample (undiluted blood DNA or 1 µg of spleen DNA). To quantify BALF5 amplicons, a standard curve with known copy numbers was generated using a double-stranded DNA gBlock containing a partial sequence of the BALF5 gene, ranging from 10^0^ to 10^8^ BALF5 copies/µl. BALF5 copy numbers in each test sample were determined by interpolating from the standard curve. Reactions were first heated to 95°C for 15 min, followed by 50 cycles of incubation at 95°C for 15 seconds and 60°C for 1 min in a QuantStudio 3 Real-Time PCR System. For blood samples, resulting BALF5 copy numbers were normalized according to each individual sample DNA concentration to present the results as EBV DNA copies/µg of DNA. Results were plotted using GraphPad Prism. For graphing purposes, all negative values based on the qPCR detection limit (<1 EBV DNA copies/µg of DNA) were assigned a value of 0.1.

### 2.25 Diagrams

All diagrams describing MVA reconstitution and stability, and animal treatment schedules were prepared using Biorender.com.

### 2.26 Quantification and statistical analysis

All statistical analyses were performed using GraphPad Prism software. For every case, the distribution of the scale variables was studied and analysis was performed using the appropriate model accordingly. In mouse MVA-EBV5-5 immunogenicity assessments, differences in IgG responses between treatment groups were assessed under a Kruskal-Wallis test. In mouse MVA-EBV5-2 immunogenicity assessments, differences in IgG responses between treatment groups on Day 49 and Day 84 were assessed via Tukey’s multiple comparison test (single pooled variance) under a mixed-effects model. In rhesus macaque immunogenicity assessments, differences in IgG responses between treatment groups were assessed via Tukey’s multiple comparison test (individual variances computed for each comparison) under a two-way ANOVA. Differences in IC50 between treatment groups for rhesus macaque neutralization assays were assessed via Dunn’s multiple comparison test under a Kruskal-Wallis test. In NSG huMice studies, differences in %hCD45+ lymphocytes between groups were assessed via Tukey’s multiple comparison test (single pooled variance) under an ordinary one-way ANOVA. Differences in EBV DNA copies (blood and spleen) between groups were assessed via Mann-Whitney test. Differences in spleen size between groups were assessed using Kruskal-Wallis test. Differences in survival between groups were assessed using Log-rank test, via comparison between pairs of groups.

## 3 Results

### 3.1 Generation and characterization of a multivalent MVA-based vaccine candidate, MVA-EBV5-2

**Figure 1:**
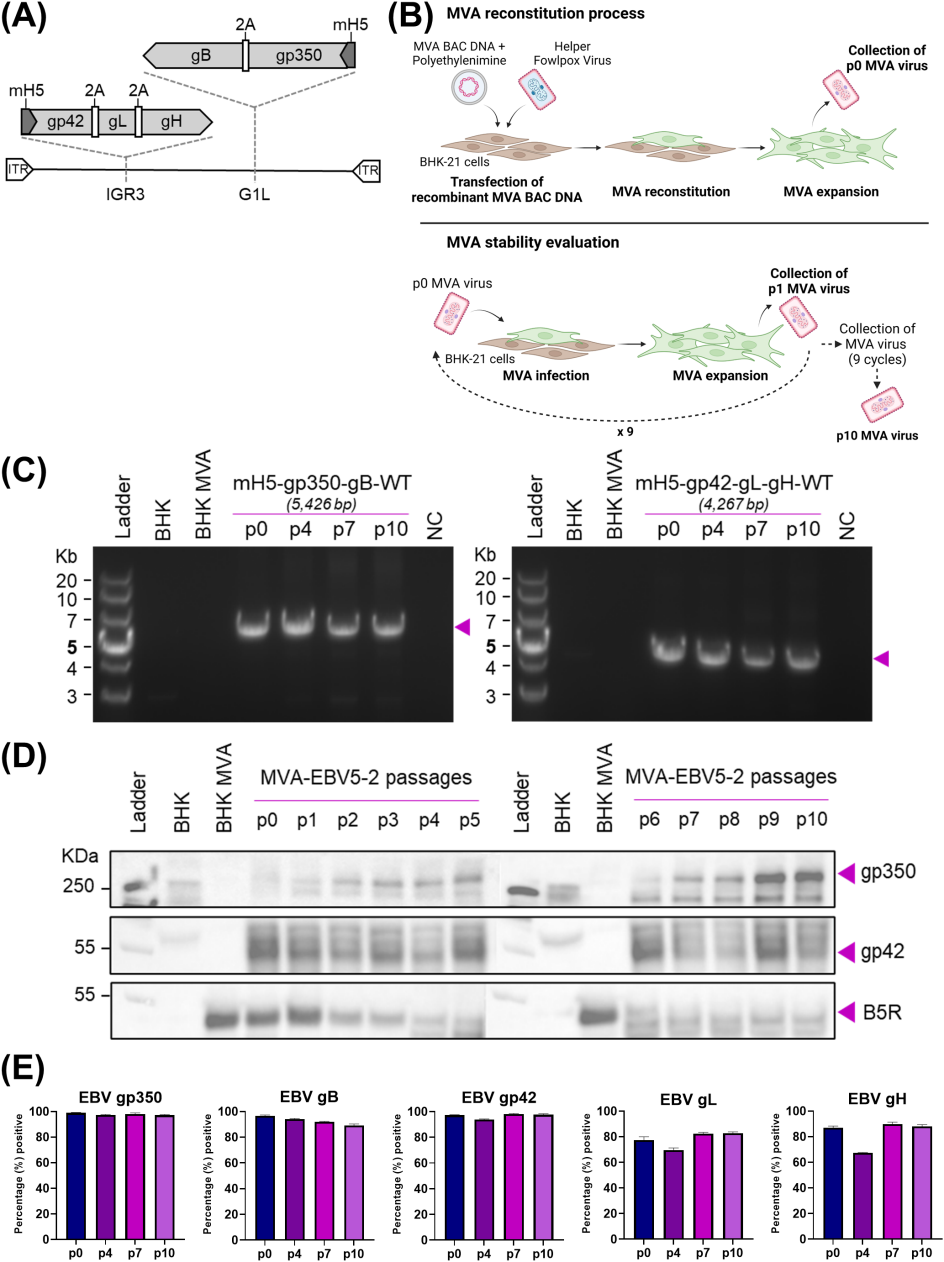
Generation and characterization of MVA-EBV5-2. **(A)** Schematic representation of MVA-EBV5-2 virus, with multivalent glycoprotein expression cassettes incorporated in the 69R/70L (G1L, gp350-gB) and 64L/65L (IGR3, gp42-gL-gH) MVA genomic sites. **(B)** Diagram depicting the reconstitution of recombinant MVA in BHK-21 cells, followed by viral serial passaging to evaluate transgene stability. **(C)** PCR amplification of the two glycoprotein expression cassettes in MVA-EBV5-2, illustrated in **(A)**, using DNA from uninfected BHK-21 cells (BHK), BHK-21 cells infected with empty MVA (BHK-MVA), or BHK-21 cells infected with serially passaged MVA-EBV5-2 virus (p0, p4, p7, and p10). NC denotes negative control, no DNA. Expected band sizes are listed above each gel and are indicated with arrows. **(D)** Binding of anti-gp350 antibody HB5, anti-gp42 antibody 15C8, and anti-B5R hybridoma supernatant to total protein from uninfected BHK-21 cells, BHK-21 cells infected with empty MVA, or BHK-21 cells infected with serially passaged MVA-EBV5-2 virus (p0–p10) was assessed using immunoblot. Bands corresponding to gp350, gp42, and B5R are indicated with arrows. *B5R is a vaccinia antigen, and is used as a loading control.* **(E)** Surface binding of the anti-gp350 monoclonal antibody (mAb) HB5, anti-gB mAb AMMO5, anti-gp42 mAb F-2-1, anti-gL mAb E1D1, and anti-gH mAb CL40 to BHK-21 cells infected with serially passaged MVA-EBV5-2 virus (p0, p4, p7, and p10) was measured using flow cytometry. Each bar represents the mean percent (%) + SD of infected cells from triplicate infections with positive signal for binding. See also **Supplementary Figures S2–S4**.

In our interest to design a multivalent EBV vaccine incorporating five EBV glycoproteins involved in viral entry of B cells and epithelial cells, we used an eGFP-expressing MVA bacterial artificial chromosome (MVA-BAC-TK) (61) to generate recombinant MVA vectors that incorporate gp350, gB, and gp42gHgL. First, we cloned a polycistronic glycoprotein expression cassette that codes for all five EBV glycoproteins in wildtype form into the MVA BAC via *en passant* mutagenesis, a homologous recombination-based cloning system (62), generating MVA-EBV5-1 (**Supplementary Figure S1A**). Similarly, we cloned two polycistronic glycoprotein expression cassettes that code for either gp42, gL, and gH (trivalent) or gp350 and gB (bivalent) in wildtype form into the MVA BAC (**Figure 1A**). During this process, we also generated MVA vectors expressing the bivalent and trivalent glycoprotein expression cassettes contained within MVA-EBV5-2, MVA-EBV-gp350-gB and MVA-EBV-gp42gLgH (**Supplementary Figure S1A**), which were subsequently used as controls in protein expression assessments. Finally, we also cloned five expression cassettes coding for each glycoprotein individually into the MVA BAC (**Supplementary Figure S1A**), generating MVA-EBV5-5. In all cases, each cassette codes for each corresponding glycoprotein(s) under the control of an mH5 promoter, a promoter that has been shown to stabilize transgenes in recombinant MVA constructs (65). To allow for individual glycoprotein release upon transcription, the glycoprotein sequences in all polycistronic cassettes are interspersed by unique self-cleaving 2A peptides (64).

To verify sequence fidelity before viral reconstitution, we performed Sanger sequencing of each expression cassette in the final recombinant MVA constructs. The resulting sequence analysis for MVA-EBV5-1 and MVA-EBV5-5 revealed full alignment with the expected sequence, except for the presence of Δ84bp (Δa.a.513-540) and Δ210bp (Δa.a.513–583) truncations in the gp350 ectodomain, respectively (data not shown). Given the presence of several 29-bp repetitive sequences in gp350, use of the *en passant* system can result in homologous recombination of these repetitive sequences, allowing the emergence of several truncated gp350 products. However, we found that these truncations, including the Δ84bp and Δ210bp truncations, occur within the gp220 splice site, which has been excluded from other recent gp350-targeting vaccines (45, 75, 76), and do not overlap with any of the previously identified gp350 neutralizing epitopes (30); thus it is unlikely that they affect antigen immunogenicity. Similar Sanger sequencing results were obtained for MVA-EBV5-2, with the expression cassettes matching the expected sequence except for the Δ210bp (Δa.a.513–583) truncation in the gp350 ectodomain (data not shown). Sanger sequencing of MVA-EBV-gp350-gB and MVA-EBV-gp42gLgH yielded results that fully matched those of MVA-EBV5-2 (data not shown). We then proceeded to transfect the DNA for each recombinant MVA construct into MVA-permissive BHK-21 cells and reconstituted the viruses using helper fowlpox virus, generating passage 0 (p0) viruses (**Figure 1B**).

Following reconstitution of p0 virus, we first assessed surface glycoprotein expression by each recombinant MVA virus in infected BHK-21 cells, via flow cytometry (**Supplementary Figures S1B**, **S2**). As expected, gp350 and gB expression were effectively detected on the surface of BHK-21 cells infected with MVA-EBV-gp350-gB, MVA-EBV5-2 and MVA-EBV5-5, while gp42, gL and gH were similarly detected on the surface of cells infected with MVA-EBV-gp42gLgH, MVA-EBV5-2 and MVA-EBV5-5. However, in comparison, gp350 and gB were detected on the surface of a lower percentage of MVA-EBV5-1-infected cells, and these cells also displayed minimal gp42 surface expression and non-detectable levels of gL or gH expression. This low level of glycoprotein expression, which could be the result of the nature of the 2A-based polycistronic expression cassette (77), prompted us to abandon MVA-EBV5-1 and focus on MVA-EBV5-2 and MVA-EBV5-5 as our potential multivalent vaccine candidates. Preliminary MVA-EBV5-5 immunogenicity experiments in BALB/c mice, when compared to MVA-EBV5-2 immunogenicity results described in the next section, led us to choose MVA-EBV5-2 as our lead vaccine candidate, as MVA-EBV5-2 immunization led to higher levels glycoprotein-specific IgG overall (**Supplementary Figure S1C**). Thus, we proceeded with further characterization of MVA-EBV5-2.

We next assessed whether the inserted glycoprotein expression cassettes in MVA-EBV5-2 were translationally active and stable after repeated viral passaging, an important quality for large-scale manufacturing of viral vector vaccines. Beginning with p0, we serially passaged MVA-EBV5-2 in BHK-21 cells ten times (65, 78) (**Figure 1B**); we collected infected cells at each passage, isolated DNA and total protein, and assessed genetic and translational stability. To assess genetic stability, we used isolated DNA as a template to PCR-amplify each expression cassette and determine the size of the amplicons at each passage. PCR amplification of the expression cassettes in p0, p4, p7, and p10 DNA resulted in amplicons of the expected size for both the bivalent and trivalent cassettes (**Figure 1C**), suggesting that sequence fidelity was maintained for up to 10 viral passages. This was confirmed by performing full genome sequencing of p0 and p10 DNA, which revealed complete alignment of the DNA to the expected sequence (data not shown). To assess translational stability, we characterized protein expression in infected BHK-21 cells. In cells infected with MVA-EBV5-2 p0–p10, we confirmed expression of gp350 and gp42 in isolated total protein using immunoblot (**Figure 1D**, vaccinia B5R antigen used as a loading control) and confirmed surface expression of all five glycoproteins using flow cytometry (**Figure 1E**, **Supplementary Figure S1**). *Note that there are no commercially available antibodies for immunoblot against gB, gH, or gL; gp42-specific antibody was recently generated and fully characterized in our laboratory (* **Supplementary Figures S3A–C** *)*. Taken together, these results demonstrate that MVA-EBV5-2 is stable, genetically and translationally, through ten viral passages.

Because MVA-EBV5-2 incorporates EBV entry glycoproteins, we then tested whether the presence of these glycoproteins could provide MVA with an alternative infection pathway into EBV-susceptible human cells. To do this, we infected the EBV-susceptible human cell lines HEK-293 and Raji with MVA-EBV5-2 or wildtype MVA, in the presence or absence of the EBV-specific neutralizing antibodies 72A1 [anti-gp350, (23)] and AMMO1 [anti-gHgL, (28)] (**Supplementary Figure S4A**). In both cell lines, the neutralizing antibodies efficiently reduced infection of EBV, which was used as a control; however, while both MVA viruses were able to infect each cell line, the antibodies did not reduce infection of either virus, suggesting that the expression of EBV entry glycoproteins did not affect the tropism of MVA towards these cells. As a final characterization step, we tested the titer stability of an MVA-EBV5-2 batch after 1 or 5 months in -80°C storage following production, using a PFU immunostaining assay, currently considered one of the traditional methods for MVA titration (68, 73) (**Supplementary Figure S4B**). Results of the assay demonstrated that MVA-EBV5-2 titers are maintained in up to 5 months in this storage condition, assuring its stability during our immunization protocols. Next, we characterized the immunogenicity of MVA-EBV5-2.

### 3.2 MVA-EBV5-2 is immunogenic in BALB/c mice

**Figure 2:**
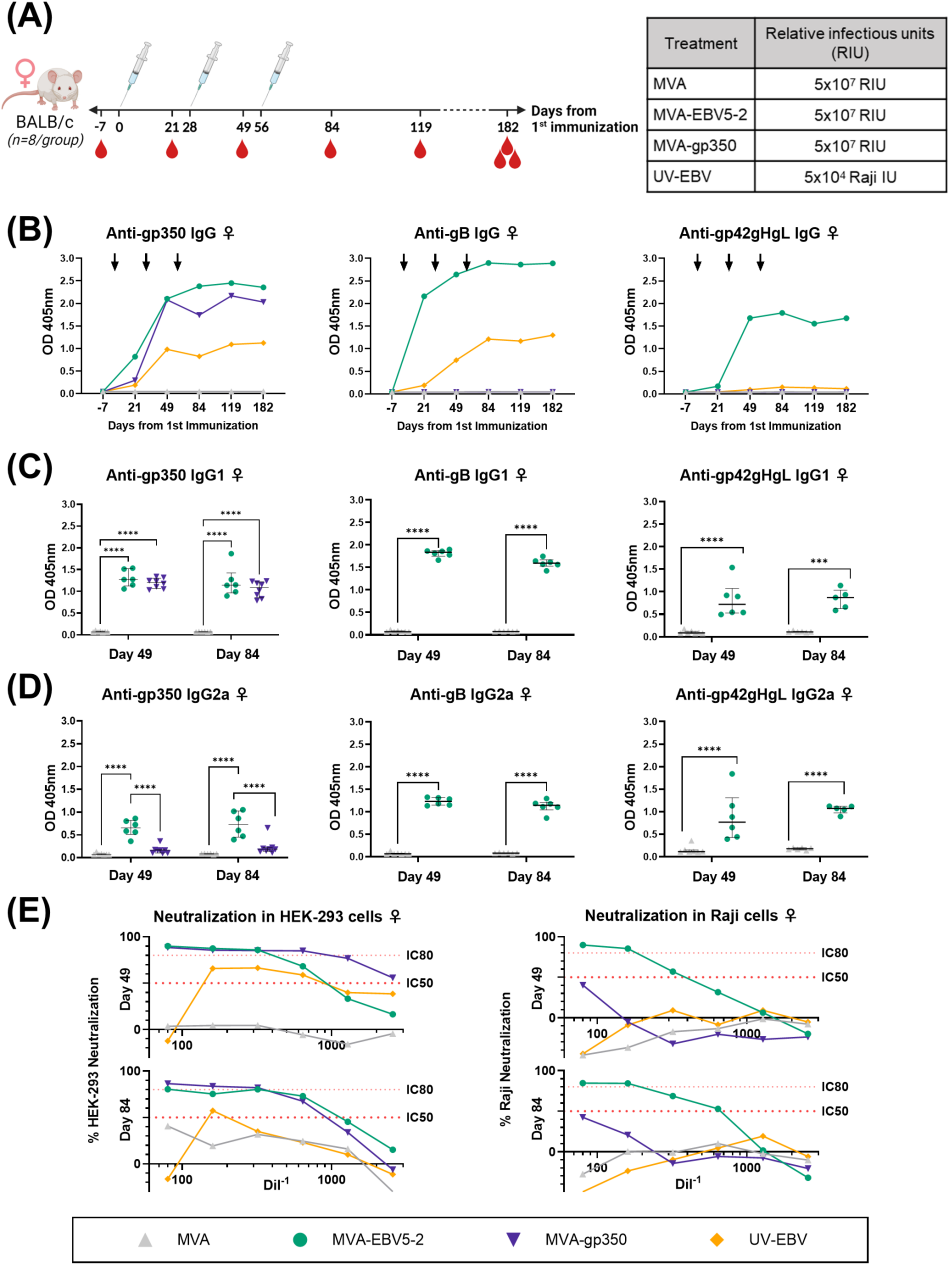
MVA-EBV5-2 is immunogenic in female BALB/c mice and elicits a neutralizing serum response against EBV infection in epithelial and Raji cells *in vitro*. **(A)** Female BALB/c mice were immunized with the indicated treatments (inset table, right) on Day 0, 28, and 56 (n=8/group). Blood was collected at the indicated timepoints (red droplets), and mice were terminally bled on Day 182. **(B)** IgG binding levels to gp350, gB, and gp42gHgL were measured using ELISA in pooled mouse serum (1/900 dilution). Each dot represents the mean of triplicate measurements, and black arrows represent immunization timepoints. **(C, D)** IgG1 **(C)** and IgG2a **(D)** binding levels to EBV gp350, gB, and gp42gHgL were measured using ELISA in individual mouse serum samples on Day 49 and 84 post-immunization (1/900 dilution; UV-EBV not shown). Each dot represents the mean of duplicate measurements for each animal, with the median and interquartile range shown for each group at each timepoint. Statistical differences were determined using Tukey’s multiple comparison test (** = p<0.01, *** = p<0.001, **** = p<0.0001). **(E)** The ability of Day 49 and 84 pooled sera to neutralize EBV infection in HEK-293 (left) and Raji (right) cells was measured via *in vitro* neutralization assays. Serum titration curves for each treatment group (% neutralization) are shown for each cell line, with the top dotted line representing 80% neutralization (IC80) and the bottom dotted line representing 50% neutralization (IC50) cut-offs.

**Figure 3:**
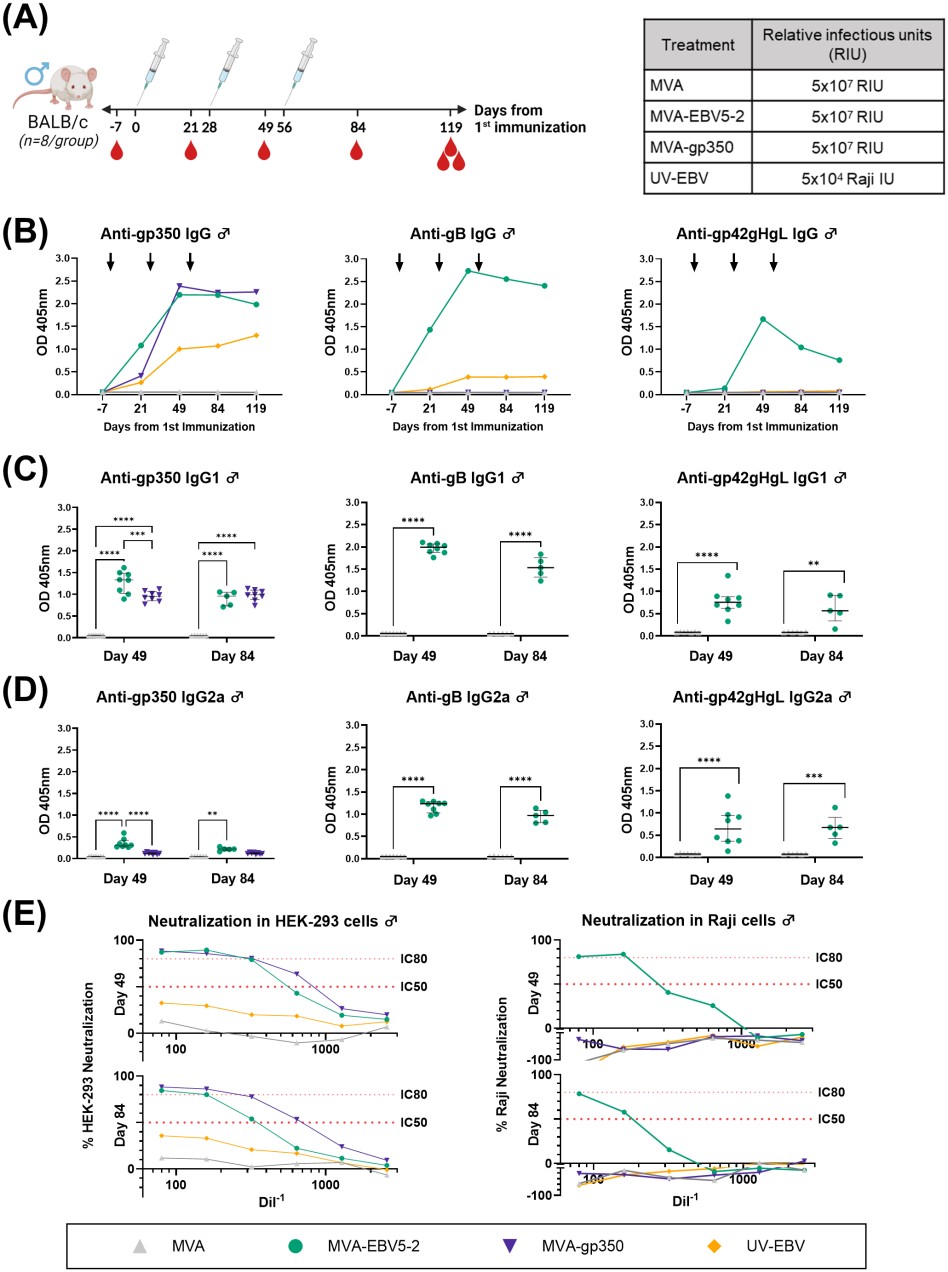
MVA-EBV5-2 is immunogenic in male BALB/c mice and elicits a neutralizing serum response against EBV infection in epithelial and Raji cells *in vitro*. **(A)** Male BALB/c mice were immunized with the indicated treatments (inset table, right) on Day 0, 28, and 56 (n=8/group). Blood was collected at the indicated timepoints (red droplets), and mice were terminally bled on Day 119. **(B)** IgG binding levels to EBV gp350, gB, and gp42gHgL were measured using ELISA in pooled mouse serum (1/900 dilution). Each dot represents the mean of triplicate measurements, and black arrows represent immunization timepoints. **(C, D)** IgG1 **(C)** and IgG2a **(D)** binding levels to EBV gp350, gB, and gp42gHgL were measured using ELISA in individual mouse serum samples on Day 49 and 84 post-immunization (1/900 dilution; UV-EBV not shown). Each dot represents the mean of duplicate measurements for each animal, with the median and interquartile range shown for each group at each timepoint. Statistical differences were determined using Tukey’s multiple comparison test (** = p<0.01, *** = p<0.001, **** = p<0.0001). **(E)** The ability of Day 49 and 84 pooled sera to neutralize EBV infection in HEK-293 (left) and Raji (right) cells was measured via *in vitro* neutralization assays. Serum titration curves for each treatment group (% neutralization) are shown for each cell line, with the top dotted line representing 80% neutralization (IC80) and the bottom dotted line representing 50% neutralization (IC50) cut-offs.

To begin assessing the immunogenicity of MVA-EBV5-2, we immunized both female (**Figure 2A**) and male (**Figure 3A**) BALB/c mice intraperitoneally with the vaccine three times at 4-week intervals. Alternatively, we immunized mice with an MVA vector that expresses gp350 (Δ210bp truncation, MVA-gp350, **Supplementary Figure S3D**) as a positive control representative of previous clinical vaccine candidates, UV-inactivated EBV (UV-EBV) as a positive control, or empty MVA virus as a negative control. We collected blood at -7, 21, 49, 84, 119, and 182 days relative to the first immunization.

To assess the levels of EBV glycoprotein-specific IgG elicited by the various treatments, we performed ELISA using the sera of immunized mice. First, we evaluated the kinetics of the humoral immune response throughout the observation period using pooled sera collected from female (**Figure 2B**) and male mice (**Figure 3B**). MVA-EBV5-2 was successful in generating IgG against gp350, gB, and the gp42gHgL complex at similar levels. In general, the level of IgG increased after the first and second dose, with limited increase after the third dose. MVA-gp350 showed similar kinetics, and as expected, elicited IgG against gp350 but not against the other four glycoproteins. UV-EBV only induced the production of IgG against gp350 and gB. As expected, no EBV-specific IgG were detected in the MVA-treated group at any timepoint.

To further characterize the humoral immune response before and after the third immunization and determine the EBV glycoprotein-specific IgG isotypes elicited by the MVA vaccines, we measured the individual mouse serum level of IgG1 (**Figures 2C**, **3C**) and IgG2a (**Figures 2D**, **3D**) by ELISA at Days 49 and 84. MVA-EBV5-2-immunized mice exhibited both IgG1 and IgG2a antibodies against all glycoproteins in both sexes; in contrast, MVA-gp350-immunized mice mainly exhibited glycoprotein-specific IgG1. We observed no increase in IgG1 or IgG2a levels after the third MVA-EBV5-2 dose (Day 84) in either sex, which together with the results of our kinetic response analysis in **Figures 2B**, **3B**, suggested that a third MVA-EBV5-2 immunization did not further boost IgG responses; thus, we reduced the number of MVA-EBV5-2 doses to two in subsequent immunogenicity experiments, which is representative of regimens used in the clinic for other MVA-based vaccines (56, 60, 79).

Finally, to determine whether the elicited antibodies exhibited EBV neutralizing activity, we performed *in vitro* neutralization assays in HEK-293 epithelial cells and Raji B cells against Akata-EBV-eGFP virus using serially diluted pooled mouse sera from samples collected on Days 49 and 84 (**Figures 2E**, **3E**). As reported in our recent systematic review, most EBV vaccine studies do not provide viral infectivity of the EBV batch used in neutralization assays (6); to ensure rigor, experimental transparency and interpretability of results, it is important that vaccine studies report the full spectrum of experimental details for neutralization assays. Here, we measured serum-free Akata-EBV-eGFP infectivity to be 13.2% and 17.6% in HEK-293 cells and 28% and 16.8% in Raji cells for female and male mice, respectively. We calculated neutralization using these values as the maximum infection rate (100% infection, or 0% neutralization). As shown, sera from female mice immunized with MVA-EBV5-2 neutralized EBV infection in both HEK-293 and Raji cells in a dose-dependent manner, better than sera from mice immunized with UV-EBV. Indeed, UV-EBV neutralizing activity was lower overall in HEK-293 cells, and virtually non-existent in Raji cells. MVA-gp350 performed similarly to MVA-EBV5-2 in HEK-293 cells but displayed much lower neutralizing activity in Raji cells. We observed similar results in male mice, although neutralizing activity was lower in all male groups overall, and the MVA-gp350 group did not display any neutralizing activity in Raji cells. Next, we set out to determine the immunogenicity of MVA-EBV5-2 in a NHP model.

### 3.3 MVA-EBV5-2 is immunogenic in rhLCV-negative rhesus macaques

**Figure 4:**
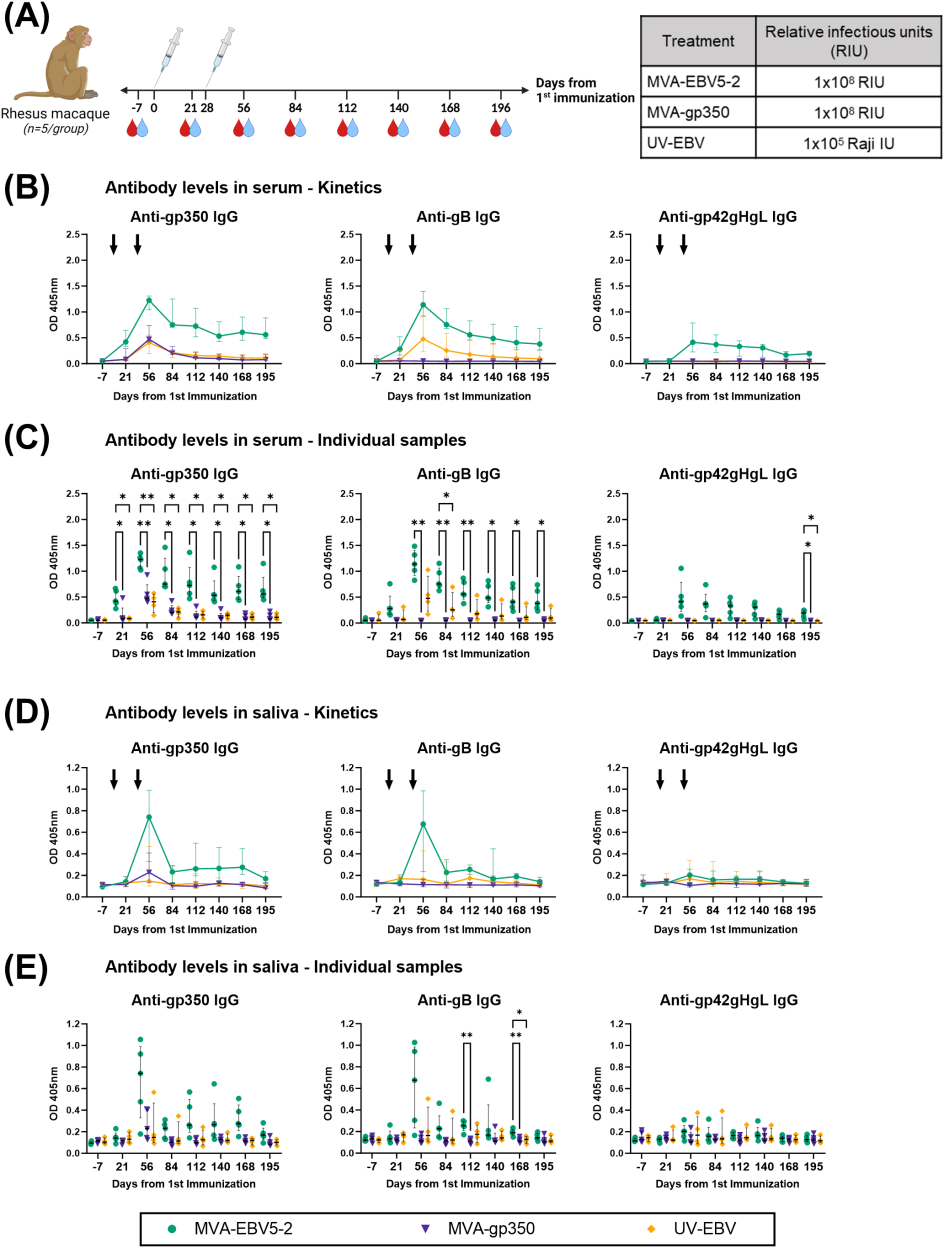
MVA-EBV5-2 is immunogenic in rhLCV-negative rhesus macaques. **(A)** rhLCV-negative female and male rhesus macaques were immunized with the indicated treatments (inset table, right) on Day 0 and Day 28 (n=5/group). Blood (red droplets) and saliva (blue droplets) were collected at the indicated timepoints. **(B, C)** IgG binding levels to EBV gp350, gB, and gp42gHgL were measured using ELISA in individual rhesus macaque serum samples (1/100 dilution). In **(B)**, the median and interquartile range are shown for each group at each timepoint, each animal was tested in duplicate. Black arrows represent immunization timepoints. In **(C)**, the same data as in B is presented but shown for individual animals, each dot representing the mean of duplicate measurements for each animal, with the median and interquartile range shown for each group at each timepoint. Statistical differences were determined using Tukey’s multiple comparison test (* = p<0.05, ** = p<0.01). **(D, E)** IgG binding levels to EBV gp350, gB, and gp42gHgL were measured using ELISA in individual rhesus macaque saliva samples (undiluted). In **(D)**, the median and interquartile range are shown for each group at each timepoint, each animal was tested in duplicate. In **(E)**, the same data as in **(B)** is presented but shown for individual animals, each dot representing the mean of duplicate measurements for each animal, with the median and interquartile range shown for each group at each timepoint. Statistical differences were determined using Tukey’s multiple comparison test (* = p<0.05, ** = p<0.01). See also **Supplementary Figure S5**.

To further validate MVA-EBV5-2 as a vaccine candidate with clinical potential, we performed an immunogenicity study in rhesus lymphocryptovirus (rhLCV)-negative rhesus macaques. Until recently (29, 45, 75, 76), most previous pre-clinical EBV vaccine studies employing NHPs did not address the fact that NHPs are hosts to pervasive EBV-homologue LCVs that can result in antigenic cross-reactivity (80), and thus affect vaccine immunogenicity assessments. To avoid this issue, the Oregon National Primate Research Center prescreened a rhesus macaque cohort for rhLCV, the rhesus-specific EBV homologue, and maintained the cohort in expanded specific-pathogen-free (SPF) conditions throughout the study to avoid rhLCV transmission. Fifteen female and male rhesus macaques were originally enrolled in the study and distributed into three treatment groups: MVA-EBV5-2, MVA-gp350, and UV-EBV (**Supplementary Figure S5A**). *Due to scarcity of rhLCV-negative rhesus macaques, we did not include an empty MVA immunization control group, but we used pre-immune serum as a negative control in all assays.* We immunized rhesus macaques twice intramuscularly (0 and 4 weeks) and collected both blood and saliva throughout the observation period (**Figure 4A**) to assess humoral immune response. To confirm rhLCV sero-negativity at the beginning of the study, we performed an rhLCV-specific ELISA using Day -7 serum from each animal (**Supplementary Figures S5B–D**); although most rhesus macaques showed no rhLCV seroreactivity, we found that one animal from the UV-EBV-immunized group (ID 33466) displayed significant levels of anti-rhLCV-gp350 IgG on par with levels in macaques from an independent non-SPF colony, and we thus excluded it from subsequent immunogenicity analyses.

To assess the levels of IgG against EBV gp350, gB, and gp42gHgL complex elicited by the vaccine, we performed ELISA using the serum and saliva of immunized rhesus macaques. In grouped and individual sera analyses from MVA-EBV5-2-immunized rhesus macaques (**Figures 4B, C**), we observed an increase of anti-gp350, -gB, and -gp42gHgL IgG, which reached maximum levels after the second dose. Indeed, we observed steady elevated levels of EBV glycoprotein-specific IgG even 6 months after the second dose. As we observed in BALB/c mice, MVA-gp350 elicited IgG against gp350 but not against the other four glycoproteins, whereas UV-EBV induced an IgG response against gp350 and gB alone. In the saliva, MVA-EBV5-2-immunized rhesus macaques (4/5) exhibited an increase in anti-gp350 and -gB IgG after the second dose; in contrast, only one animal in the UV-EBV-immunized group exhibited such an increase (**Figures 4D, E**). In the MVA-gp350 group, only one animal exhibited an increase in anti-gp350 IgG after the second dose. No animals in any group exhibited an increase in anti-gp42gHgL IgG. To assess whether the vaccine elicited mucosal antibodies in the saliva, we also attempted to measure EBV glycoprotein-specific IgA, but did not detect it in any immunized rhesus macaque (not shown); however, in the absence of a proper rhesus IgA positive control, these results should be interpreted with caution.

**Figure 5:**
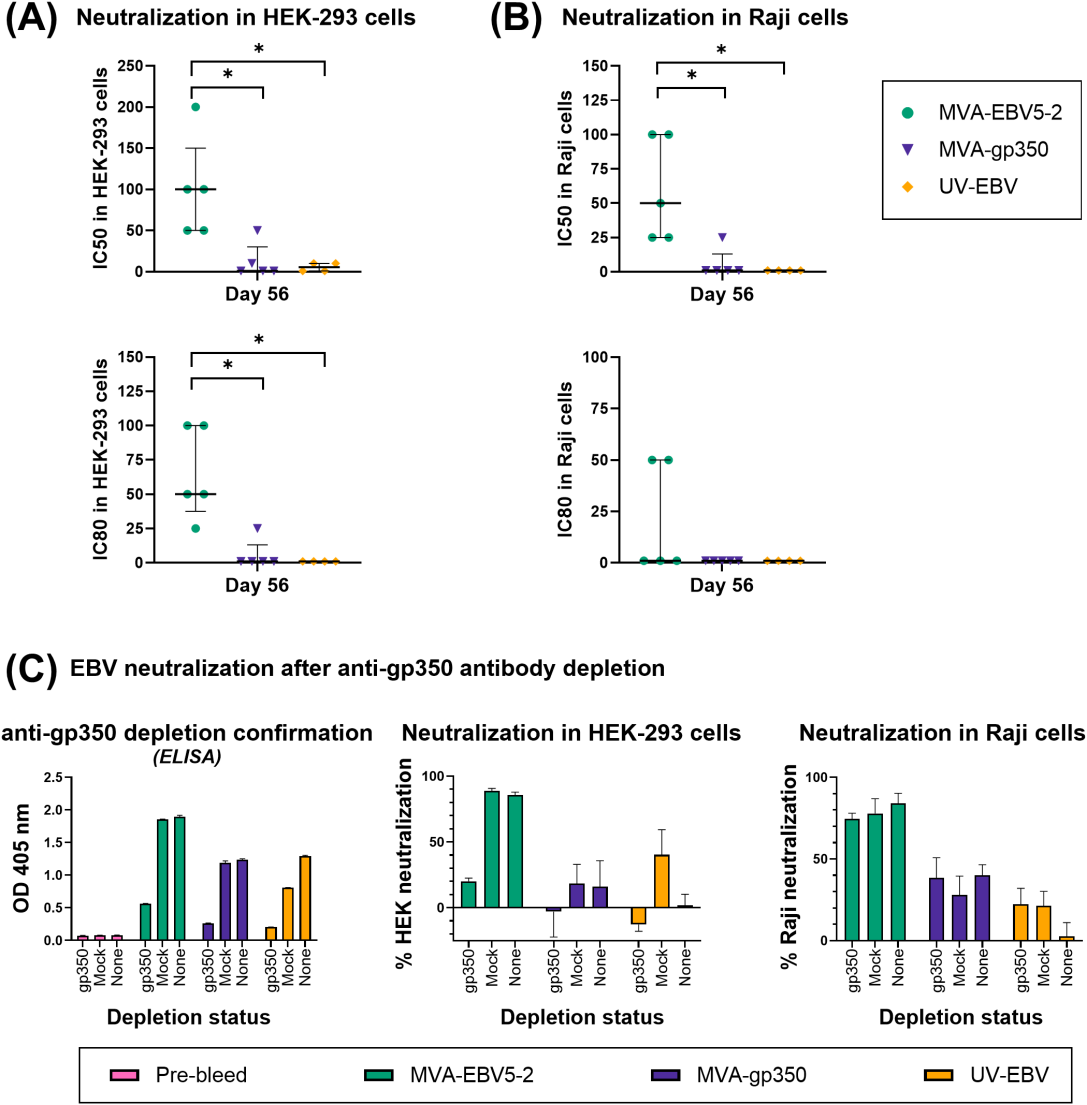
The sera of MVA-EBV5-2-immunized rhesus macaques neutralize EBV infection in epithelial and B cells *in vitro*. **(A, B)** The ability of serially diluted Day 56 sera from individual rhesus macaques in **Figure 4** to neutralize EBV infection in HEK-293 **(A)** and Raji **(B)** cells was measured via *in vitro* neutralization assay. Each dot represents the reciprocal of the last dilution at which ≥50% (top panel) or ≥80% (bottom panel) neutralization was achieved for each animal (IC50 and IC80, respectively), as compared to the level of infection in the presence of Day -7 serum for each corresponding animal. The median and interquartile range is shown for each group. Statistical differences were determined using Kruskal-Wallis test (* = p<0.05). **(C)** The ability of Day 56 pooled sera from rhesus macaques in **Figure 4** to neutralize EBV infection before and after anti-gp350 antibody depletion was measured via *in vitro* neutralization assays. Pooled sera at a 1/12.5 dilution from each treatment group was incubated with a gp350-coated nitrocellulose membrane, and before neutralization assays, IgG binding levels to EBV gp350 (1/50 sera dilution) were measured using ELISA (first panel) in depleted sera (gp350), sera incubated with a BSA-blocked nitrocellulose membrane (mock), or undepleted sera (none); bar graphs represent the mean + SD of duplicate measurements for each group. Anti-gp350 antibody-depleted sera and control sera was subsequently used in HEK-293 (middle panel) and Raji (right panel) cell neutralization assays; bar graphs represent the mean + SD neutralization of triplicate measurements for each group, at a 1/50 sera dilution. See also **Supplementary Figures S6, S7**.

To determine the neutralizing potential of serum antibodies elicited by MVA-EBV5-2 in rhesus macaques, we performed *in vitro* neutralization assays in both HEK-293 epithelial cells (**Figure 5A**, **Supplementary Figure S6A**) and Raji B cells (**Figure 5B**, **Supplementary Figure S6B**) using serially diluted individual serum samples collected on Day 56. We measured serum-free Akata-EBV-eGFP infectivity as 25.0% and 5.3% for HEK-293 and Raji cells, respectively. To eliminate any serum effect, we calculated IC50 and IC80 values based on the neutralization achieved using sera collected on Day -7 (Pre-immune) versus Day 56 from the same rhesus macaque at the same dilution. In both HEK-293 and Raji assays, serum samples from MVA-EBV5-2-immunized animals outperformed samples from MVA-gp350 and UV-EBV-immunized animals in neutralizing EBV infection, confirming that MVA-EBV5-2 elicited higher levels of neutralizing antibodies than these controls.

Given that gp350 is the main target of neutralizing antibodies against EBV B cell infection in EBV-seropositive individuals (29), we determined the contribution of gp350-specific antibodies to the neutralizing activity of immune rhesus macaque sera from the different treatment groups. To achieve this, we pooled Day 56 immune sera for each rhesus macaque group and incubated each serum pool with nitrocellulose membranes either coated with gp350 protein and blocked with bovine serum album (BSA) (gp350 depletion), or only blocked with BSA (mock depletion). After verifying that gp350-specific antibodies were depleted from the sera pools (**Figure 5C**, left panel), we proceeded to repeat neutralization assays using depleted sera, mock-depleted sera, and undepleted sera in both HEK-293 and Raji cells (**Figure 5C**, middle and right panel), measuring serum-free Akata-EBV-eGFP infectivity as 16.5% and 4.6% for HEK-293 and Raji cells, respectively. In MVA-EBV5-2 sera, most of the neutralizing activity was lost in HEK-293 cells after anti-gp350 antibody depletion; in Raji cells however, there was almost no reduction in neutralizing activity when comparing depleted versus undepleted sera. MVA-gp350 sera lost all neutralizing activity in HEK-293 cells after depletion, but neutralizing activity in Raji cells was not affected. Interestingly, UV-EBV sera gained neutralizing activity in HEK-293 cells when mock-depleted, but this activity was lost upon depleting anti-gp350 antibodies; in contrast, in Raji cells, both depletions resulted in an increased neutralizing activity. We attribute this effect in UV-EBV sera to interference from BSA-specific antibodies elicited by UV-EBV immunization. Indeed, mock depletion with a BSA-coated membrane resulted in a decreased OD when compared to undepleted sera during the anti-gp350 antibody depletion confirmatory ELISA (**Figure 5C**, left panel), which was performed with BSA as blocking agent. We confirmed this anti-BSA immunity in both mouse and rhesus UV-EBV groups in further ELISAs by comparing serum antibody binding to BSA between immune and Pre-immune sera, but did not observe it in the MVA groups (not shown). Considering that anti-gp350 antibody depletion of MVA-EBV5-2 sera did not significantly reduce neutralization in Raji cells, we further studied the contribution of the different target glycoproteins by performing additional depletion experiments (**Supplementary Figure S7**). After confirming the depletion of anti-gp350, -gB and -gp42gHgL antibodies in MVA-EBV5-2 sera by ELISA (**Supplementary Figure S7**, top panel), we repeated the neutralization assays in both HEK-293 and Raji cells (**Supplementary Figure S7**, bottom panel), reaching a serum-free Akata-EBV-eGFP infectivity of 11.7% and 30.4% in each cell line respectively. Results confirmed that most of the neutralizing activity in HEK-293 cells is due to the presence of anti-gp350 antibodies, while in Raji cells this effect is mainly associated with anti-gp42gHgL antibodies. Next, we assessed the protection against infection provided by MVA-EBV5-2-elicited antibodies *in vivo*.

### 3.4 Passive transfer of MVA-EBV5-2-immunized rhesus macaque sera protects humanized mice against EBV infection

**Figure 6:**
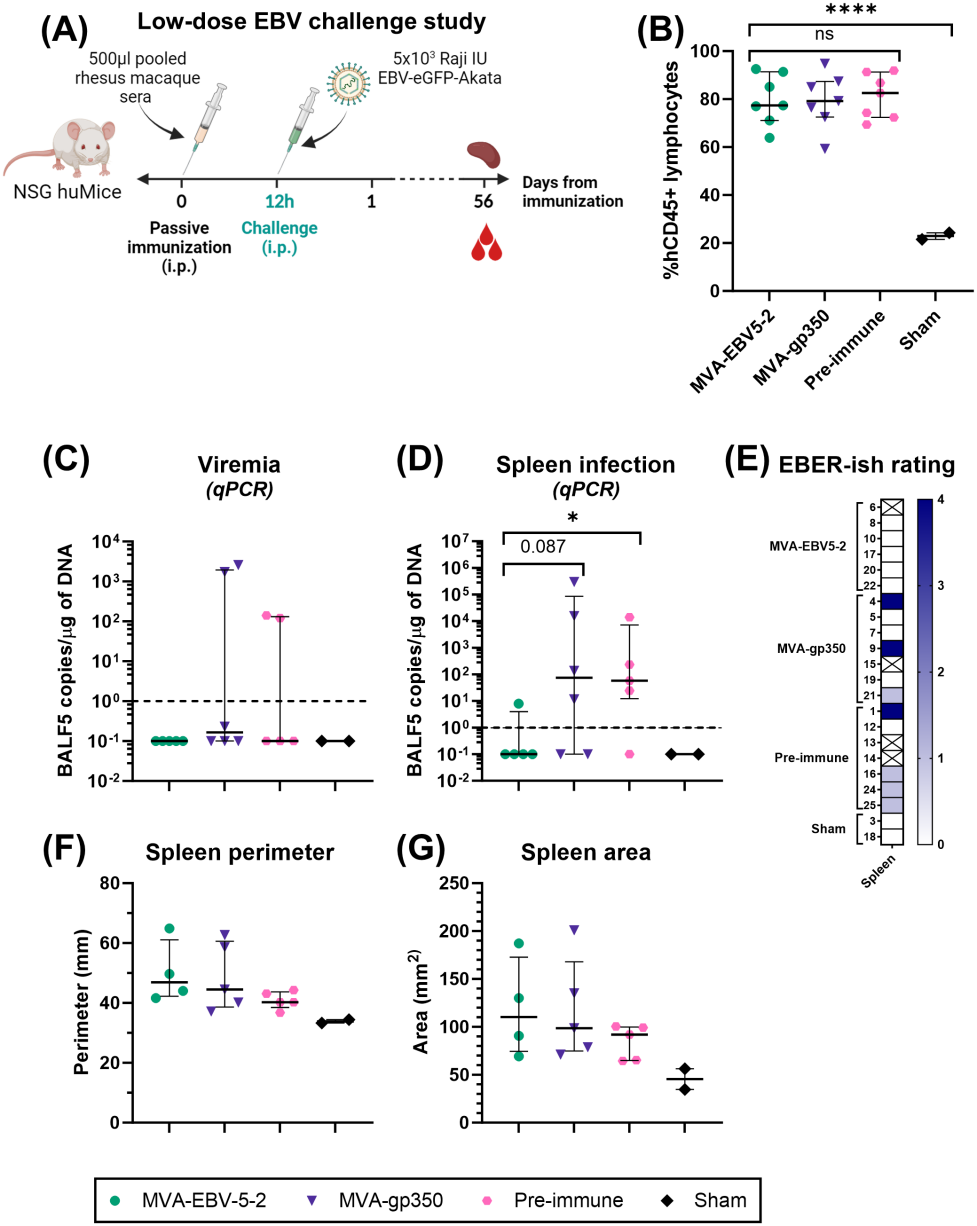
MVA-EBV5-2-elicited antibodies protect NSG huMice from low-dose EBV challenge better than antibodies elicited by a monovalent gp350 vaccine. **(A)** Female and male NSG huMice were passively immunized intraperitoneally (i.p.) with 500 µl of Day 56 MVA-EBV5-2-immune sera, Day 56 MVA-gp350-immune sera, or Pre-immune sera from rhesus macaques in **Figure 4** (n=7/group). Twelve hours post-immunization, mice were challenged with 5x10^3^ Raji IU Akata-EBV-eGFP i.p., then monitored for 56 days, after which animals were euthanized and tissues were collected. An additional group of mice (n=2) were neither immunized nor challenged to serve as a Sham control group. **(B)** Percent (%) hCD45+ lymphocytes was assessed using flow cytometry in collected circulating blood of NSG mice reconstituted with hCD34+ cells, 12 weeks post-engraftment and prior to immunization. Shown are individual mouse measurements after allocation into treatment groups using a balanced allocation logarithm. *Note that the two mice in the Sham group had low percentages of hCD45+ lymphocytes, and were not included in the balanced allocation algorithm to prioritize NSG huMice with high levels of hCD45+ lymphocytes in experimental groups*. The median and interquartile range are shown for each group. Statistical differences were determined using Tukey’s multiple comparison test (ns = not significant, **** = p<0.0001). **(C, D)** Viral DNA in the peripheral blood **(C)** or spleens **(D)** of NSG huMice was quantified via qPCR after euthanization. Each dot represents the mean of individual mouse measurements, each animal was tested in duplicate. The median and interquartile range are shown for each group. The dashed line represents the limit of detection. Statistical differences were determined using Mann-Whitney test (* = p<0.05). **(E)** The presence of EBER in the spleens of NSG huMice was assessed via *in situ* hybridization after euthanization. Shown is the EBER spleen staining score for each mouse on a grading scale of 0–4. *Crossed-out rectangles represent non-evaluable mice that succumbed before sample collection.* **(F, G)** Perimeter **(F)** and area **(G)** of collected spleens from NSG huMice were quantified using IC Measure software. Shown are individual mouse measurements with the median and interquartile range shown for each group. Statistical differences were assessed using Kruskal-Wallis test, but no differences were detected. See also **Supplementary Figures S8–S10** and **Supplementary Table S2**.

**Figure 7:**
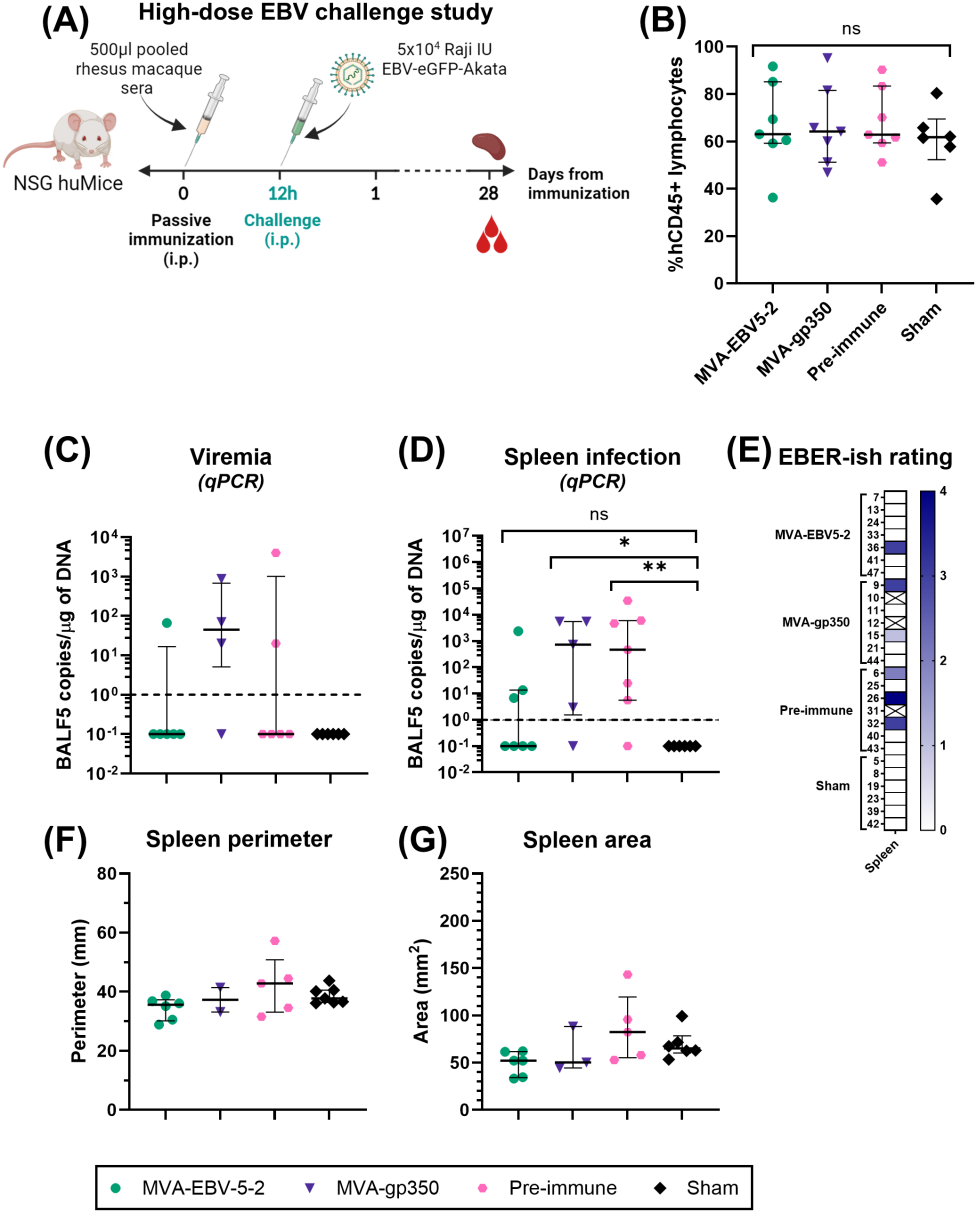
MVA-EBV5-2-elicited antibodies protect NSG huMice from high-dose EBV challenge better than antibodies elicited by a monovalent gp350 vaccine. **(A)** Female and male NSG huMice were passively immunized intraperitoneally (i.p.) with 500 µl of Day 56 MVA-EBV5-2-immune sera, Day 56 MVA-gp350-immune sera, or Pre-immune sera from rhesus macaques in **Figure 4** (n=7/group). Twelve hours post-immunization, mice were challenged with 5x10^4^ Raji IU Akata-EBV-eGFP i.p., then monitored for 28 days, after which animals were euthanized and tissues were collected. An additional group of mice (n=6) were neither immunized nor challenged to serve as a Sham control group. **(B)** Percent (%) hCD45+ lymphocytes was assessed using flow cytometry in collected circulating blood of NSG mice reconstituted with hCD34+ cells, 12 weeks post-engraftment and prior to immunization. Shown are individual mouse measurements after allocation intro indicated treatment groups using a balanced allocation logarithm. The median and interquartile range are shown for each group. Statistical differences were determined using Tukey’s multiple comparison test (ns = not significant). **(C, D)** Viral DNA in the peripheral blood **(C)** or spleens **(D)** of NSG huMice was quantified via qPCR after euthanization. Each dot represents the mean of individual mouse measurements, each animal was tested in duplicate. The median and interquartile range are shown for each group. The dashed line represents the limit of detection. Statistical differences were determined using Mann-Whitney test (ns = not significant, * = p<0.05, ** = p<0.01). **(E)** The presence of EBER in the spleens of NSG huMice was assessed via *in situ* hybridization after euthanization. Shown is the EBER spleen staining score for each mouse on a grading scale of 0–4. *Crossed-out rectangles represent non-evaluable mice that succumbed before sample collection.* **(F, G)** Perimeter **(F)** and area **(G)** of collected spleens from NSG huMice were quantified using IC Measure software. Shown are individual mouse measurements with the median and interquartile range shown for each group. Statistical differences were assessed using Kruskal-Wallis test, but no differences were detected. See also **Supplementary Figures S8–S10** and **Supplementary Table S3**.

To assess the ability of MVA-EBV5-2-generated antibodies to prevent EBV infection *in vivo*, we performed vaccine efficacy studies in immunodeficient NOD scid gamma mice engrafted with human CD34+ hematopoietic stem cells (NSG huMice) (**Figures 6A**, **7A**). Before beginning the studies, we assessed the mean percentage of human CD45-positive (%hCD45+) lymphocytes in the circulating blood of each mouse, then used a balanced allocation randomization algorithm to ensure %hCD45+ expression was balanced across mice distributed to all treatment groups (**Figures 6B**, **7B**). Individual mouse information and respective %hCD45+ lymphocyte values assessed by flow cytometry (**Supplementary Figure S8A**) are listed in **Supplementary Tables S2** and **S3**. We performed two independent studies in which we passively immunized female and male NSG huMice with sera from immunized rhesus macaques, then challenged them with either a low or a high dose of EBV. Mice in the MVA-EBV5-2 and MVA-gp350 groups were passively immunized with Day 56 pooled serum from immunized rhesus macaques described in **Figure 4**. Mice in the Pre-immune group were passively immunized with Day -7 pooled serum from all rhesus macaque groups (negative control). After 12 hours, the immunized mice were challenged with either 5x10^3^ Raji infectious units (IU) (low dose, **Figure 6A**) or 5x10^4^ Raji IU (high dose, **Figure 7A**) of EBV. Mice in the Sham group were neither passively immunized with rhesus macaque sera nor challenged with EBV. The challenge timepoint was chosen based on a preliminary serum kinetics study (**Supplementary Figure S8B**), in which we determined that rhesus glycoprotein-specific IgG reached a maximum and stable level in the sera of NSG huMice by 12 hours post-intraperitoneal passive immunization (**Supplementary Figure S8C**). Moreover, when we performed an intraperitoneal inspection of the mice at each final timepoint, we found no non-absorbed rhesus macaque sera after 12 hours. In both studies, we assessed EBV infection in available samples harvested upon death, whether in mice that completed the observation period, or mice that either perished early or were euthanized early due to poor health (**Supplementary Tables S2**, **S3**); however, not all mice had evaluable samples at the end of the study.

To assess infection outcomes in the low-dose EBV challenge study, we monitored mice for 56 days, then euthanized them, collected blood and spleens (**Figure 6A**), and measured blood viremia and splenic infection by performing quantitative PCR (qPCR) against the EBV BALF5 gene. We also assessed splenic infection by performing EBV-encoded RNA (EBER) *in situ* hybridization. No mice in the MVA-EBV5-2 group (0/5) exhibited viremia, in contrast to detectable viremia in the MVA-gp350 (2/6) and Pre-immune (2/5) groups (**Figure 6C**). Similarly, only one mouse in the MVA-EBV5-2 group (1/5) displayed splenic infection by qPCR, with a low number of EBV DNA copies, in contrast to higher infection levels in the MVA-gp350 (4/6) and Pre-immune (4/5) groups (**Figure 6D**). Consistent with this, no mice displayed an EBER-positive signal in the spleen in the MVA-EBV5-2 group (0/6), in contrast to greater EBER-positivity in the MVA-gp350 (3/6) and Pre-immune (4/6) groups (**Figure 6E**, **Supplementary Figure S9A**). We also compared spleen size and morphology between the groups but found no size differences or gross morphologies indicative of tumors (**Figures 6F, G**, **Supplementary Figure S10A**). Similarly, we found no differences in survival rate between the groups (**Supplementary Figure S8D**, left).

In the high-dose EBV challenge study, in which we used a 10-fold higher EBV dose compared to the low-dose cohort, we reduced the length of the study from 56 days to 28 days to ensure mouse welfare (**Figure 7A**), guided by our periodic health evaluation of the animals, then evaluated blood viremia and splenic infection as above. We detected viremia in only one mouse in the MVA-EBV5-2 group (1/6), in contrast to the MVA-gp350 (3/4) and Pre-immune (2/6) groups (**Figure 7C**). In the spleen, we detected a greater number of EBV DNA-positive mice in all treatment groups when compared to mice in the low-dose cohort (**Figure 7D** versus **Figure 6D**), likely owing to the higher dose of EBV used. However, even at this high dose, we observed fewer mice with splenic infection in the MVA-EBV5-2 group (3/7), compared to the MVA-gp350 (4/5) and Pre-immune (6/7) groups (**Figure 7D**). As in the low-dose study, splenic EBER staining detected fewer EBV-positive mice in the MVA-EBV5-2 group (1/7) compared to the MVA-gp350 (2/6) and Pre-immune (3/6) groups (**Figure 7E**, **Supplementary Figure S9B**). The discrepancy between splenic qPCR vs. EBER staining for EBV detection prompted us to compare the results of the two techniques (**Supplementary Figure S8E**). We found that between 2x10^1^ and 6x10^2^ EBV copies/ug DNA as detected by qPCR, EBER staining can fail to detect EBV-positive samples, with no detection below that range. In contrast, qPCR successfully detected as few as 3 EBV copies/ug DNA, suggesting that qPCR can detect EBV infection with a higher sensitivity; future studies should therefore use EBER staining as a complimentary technique to qPCR in detecting EBV infection. Regardless, EBER staining results support our previous observations that fewer MVA-EBV5-2 mice displayed splenic infection than in the control groups. Regarding spleen size and morphology, we did not find any size differences or observe gross morphologies indicative of tumors (**Figures 7F, G**, **Supplementary Figure S10B**). For survival, we only detected a difference between the MVA-gp350 and Sham group (**Supplementary Figure S8D**, right), but whether this effect is real and not due to other inherent health issues in the mice is unclear.

## 4 Discussion

EBV infection and its associated diseases remain a significant health burden, with no licensed prophylactic vaccine available despite four decades of vaccine research. In response to EBV vaccine efforts shifting toward multivalent approaches (6), and building upon our previous multivalent EBV vaccine (48), we designed an MVA-based vaccine that incorporates five EBV glycoproteins important for viral entry into diverse cell types: gp350, gB, and gp42gHgL complex (**Figures 1A, B**). After confirming that our vaccine, MVA-EBV5-2, was genetically and translationally stable over 10 viral passages (**Figures 1C–E**), we confirmed its immunogenicity and efficacy via three animal models (**Figures 2**–**7**): BALB/c mice and rhesus macaques (immunogenicity) and NSG huMice (efficacy).

In immunized BALB/c mice and rhesus macaques, MVA-EBV5-2 elicited a robust serum IgG response against all target EBV glycoproteins (**Figures 2A**, **3A**, **4B, C**). When we further dissected the IgG response in mice, we found that MVA-EBV5-2 elicited glycoprotein-specific IgG of both IgG2a and IgG1 subtypes, as opposed to our MVA-gp350 control, which primarily elicited IgG1 (**Figures 2C, D**, **3C, D**). This suggests that MVA-EBV5-2 elicited both Th1- and Th2-type immune responses; however, in the absence of additional experiments, this assumption should be taken with caution. A balanced Th1/Th2 response implies that the vaccine can elicit both strong humoral immunity against EBV virions, as well as cytotoxic cellular immunity that could target EBV-infected cells should the virus escape antibody-mediated clearance (81, 82), and we plan to test this in future experiments. Additionally, in rhesus macaques, we showed that MVA-EBV5-2-elicited IgG can be detected in the saliva of immunized animals (**Figures 4D, E**), which has not been reported in previous EBV vaccine studies. Given that the vaccine was administered intramuscularly and was not targeted to the mucosa (83, 84), the observed Ig were most likely not locally produced and instead leaked into the oral cavity from the blood through the gingival crevices (85). Despite the potential protection that the produced IgG could provide in the oral cavity, identifying alternative immunization routes that optimally target the mucosa will be key in further enhancing the salivary humoral response against this orally transmitted virus.

Our group and the Cohen group have consistently shown that immunization with multivalent vaccines elicits antibodies with higher *in vitro* neutralizing activity than immunization with monovalent gp350-based vaccines (29, 45, 48). In the mouse experiments of this study, both MVA-EBV5-2 and MVA-gp350 sera outperformed UV-EBV sera in HEK-293 cell neutralization assays (**Figures 2E**, **3E**), with MVA-gp350 displaying slightly higher levels of neutralizing activity than MVA-EBV5-2 in Day 49 and Day 84 serum for female (**Figure 2E**) and male (**Figure 3E**) mice, respectively. EBV infection of HEK-293 cells is dependent on CR2/CD21 (13), a gp350 cellular receptor, and is thus expected to be susceptible to gp350-specific antibody-mediated EBV neutralization (47, 48). Other epithelial cell lines of clearer origin have been used for EBV neutralization, but either our group hasn’t yet secured access to them [HNE1 (31, 34, 86)], they have been engineered to artificially express CR2 to enable EBV infection [SVKCR2 (6, 45, 87, 88)], or in our hands result in very low infectivity [1-2% infection, AGS (6)] that complicates and reduces the robustness of neutralization analysis and affects reproducibility; thus, we have consistently used HEK-293 cells for EBV neutralization in our work, as have others (45, 47, 48, 89). In Raji neutralization, MVA-EBV5-2 sera outperformed MVA-gp350 sera in both female (**Figure 2E**) and male (**Figure 3E**) mice, suggesting that additional antibodies could be involved in neutralization of this cell line, as levels of serum gp350-specific IgG were similar between MVA-EBV5-2- and MVA-gp350-immunized mice. In the rhesus macaque experiment, MVA-EBV5-2 sera outperformed both MVA-gp350 and UV-EBV sera in neutralization assays of both cell lines (**Figures 5A, B**). Depletion of gp350-specific antibodies from pooled rhesus macaque sera (**Figure 5C**) resulted in a stark decrease in HEK-293 neutralizing activity in the MVA-EBV5-2 sample, and a complete abrogation in the MVA-gp350 sample, in line with our previous observations that HEK-293 cells are sensitive to gp350-dependent neutralization. Interestingly, gp350-specific antibody depletion did not reduce the neutralizing activity of either MVA-gp350 or MVA-EBV5-2 sera in Raji cells. In the case of MVA-gp350 sera, we did not expect to see a lack of effect upon antibody depletion, but our result could be due to incomplete antibody depletion. In the case of MVA-EBV5-2, our observations suggested that other antibodies, namely gB- or gp42gHgL-specific antibodies, could be playing a major neutralizing role in this cell line, as has been previously observed (28, 29, 31, 35, 86, 87, 89). To explore this possibility, we performed further depletion experiments in MVA-EBV5-2 sera (**Supplementary Figure S7**), which confirmed our previous observations regarding anti-gp350 antibodies in both HEK-293 and Raji cells, and revealed that the major MVA-EBV5-2 neutralizing compartment against EBV Raji infection were anti-gp42gHgL antibodies. We did not observe any effects upon anti-gB antibody depletion in either cell line. Finally, the UV-EBV sera displayed a curious effect dependent on anti-BSA antibodies. To our surprise, we found that UV-EBV immunization elicited anti-BSA immunity, most likely originating from the fetal bovine serum used in the culture of the EBV producer cell lines. The resulting BSA antibodies appear to enhance infection, as incubation of UV-EBV sera with BSA-coated membrane (mock depletion) resulted in enhanced neutralization in both HEK-293 and Raji cells (**Figure 5C**), and in neutralization assays in female mice, highly concentrated UV-EBV sera dilutions resulted in increased infection (**Figure 2E**). Taking mock-depleted sera as the comparator, gp350-specific antibody depletion in UV-EBV sera completely abrogated neutralizing activity in HEK-293 cells, as we observed for MVA-gp350 sera. In contrast, in Raji cells, we did not observe any reduction in neutralizing activity, similar to MVA-EBV5-2 sera, again suggesting the involvement of non-gp350-specific antibodies in neutralization.

In the absence of a readily accessible and truly representative *in vivo* EBV infection model, humanized mice are becoming increasingly popular for testing antibody and vaccine efficacy against EBV infection (31, 33–35, 45, 46, 86, 87, 90–92). Here, we used NSG huMice (93, 94) to test the protective efficacy of passively immunized rhesus macaque MVA-EBV5-2-immune sera against two dose levels of EBV (**Figures 6**, **7**). In both studies, our *in vivo* results were consistent with our *in vitro* results, as MVA-EBV5-2-immune sera provided superior protection against B cell infection than did MVA-gp350-immune sera. To date, our studies are the first to compare protection between a multivalent vaccine and a monovalent gp350-based vaccine *in vivo*. Previous EBV vaccines assessed in humanized mouse studies include gHgL-based nanoparticles (87), gp350-based nanoparticles in combination with either gHgL- or gp42gHgL-based nanoparticles (45), gB-based nanoparticles (86), and either gHgL or trivalent gB protein (46). The nanoparticle studies, by Malhi et al. (87), Wei et al. (45) and Sun et al. (86), used purified IgG from immunized animals rather than immune sera for passive immunization experiments, whereas the protein study, by Cui et al. (46), was the only study before our own that used immune sera. In all four cases, the experimental vaccines reduced infection when compared to non-immune controls. Although not a vaccine study, Singh et al. (91) compared the protective efficacy of the gHgL-specific neutralizing antibody, AMMO1, to the gp350-specific neutralizing antibody, 72A1, and found superior protection against infection in humanized mice passively immunized with AMMO1. Other antibody studies in humanized mice have also demonstrated the neutralizing potential of non-gp350-specific antibody glycoprotein targets (31, 33–35). Because of differences in study design and humanized mouse models used, it is difficult to directly compare our results with these previously published studies, but our combined results provide support for the inclusion of multiple glycoprotein targets in an EBV vaccine.

Of note, no previous antibody or vaccine study in humanized mice, including our own, has resulted in sterilizing immunity against EBV. However, it is important to consider the caveats of the humanized mouse model. Humanized mice can only recapitulate human B cell infection, and as such, require viral inoculation through non-natural routes at high viral doses to enable successful infection (94, 95). In addition, for vaccine studies, these mice cannot be directly immunized and require passive immunization of immune sera or antibodies due to their inability to mount effective humoral immune responses. Thus, humanized mouse studies, although helpful in providing important insights regarding the neutralizing potential of a given antibody or vaccine, cannot provide a complete assessment of its efficacy. In non-human settings, this will only be possible through the use of alternate animal models, such as the common marmoset model, previously reported to be susceptible to oral EBV infection (96, 97), or the use of the rhLCV infection model in rhesus macaques as an EBV surrogate infection model, which recapitulates most features of human EBV infection (98). Recently, three new glycoprotein-based prophylactic EBV vaccine clinical trials have been registered, testing a multimeric gp350-based nanoparticle approach (99, 100), and a multivalent mRNA-based approach (101). These trials are expected to provide new insights as the first studies to test multimeric and multivalent EBV vaccine approaches in human settings, and may contribute to establishing true correlates of immune protection.

In summary, we demonstrated that a multivalent MVA-based vaccine targeting multiple EBV entry glycoproteins is immunogenic in mice and rhesus macaques and provides superior protection to NSG huMice against EBV challenge when compared to a monovalent gp350-based vaccine. Our results are in agreement with previous studies that support the use of diverse entry glycoproteins in EBV vaccine design, and we foresee that future multivalent vaccine studies involving more accurate animal models of EBV infection, coupled with ongoing and future human clinical trials, will yield critical information to support the licensure of an effective EBV vaccine.

## Data Availability

The raw data supporting the conclusions of this article will be made available by the authors, without undue reservation.
