# Supplementary material for: Multivalent MVA-vectored vaccine elicits EBV neutralizing antibodies in rhesus macaques that reduce EBV infection in humanized mice
Source: Front Immunol. 2024 Sep 13;15:1445209. doi: 10.3389/fimmu.2024.1445209 (PMC11427267; doi:10.3389/fimmu.2024.1445209)
Supplement: Supplementary file 1 [file DataSheet1.pdf]

Supplementary Material

Supplementary Table 1 (Table S1). Detailed material and resource information.

REAGENT or RESOURCE	SOURCE	IDENTIFIER
Antibodies		
F-2-1 (mouse anti-gp42)	Dr. Lindsey Hutt-Fletcher and Dr. Rona Scott, (1)	N/A
CL40 (mouse anti-gH)	Dr. Lindsey Hutt-Fletcher and Dr. Rona Scott, (2)	N/A
E1D1 (mouse anti-gL)	Dr. Lindsey Hutt-Fletcher and Dr. Rona Scott, (3)	N/A
HB5 (mouse anti-gp350)	(4)	N/A
AMMO5 (human anti-gB)	Dr. Andrew McGuire, (5)	N/A
72A1 (mouse anti-gp350)	(4, 6)	N/A
AMMO1 (human anti-gHgL)	(5)	N/A
15C8 (mouse anti-gp42)	This paper	N/A
19C2 hybridoma supernatant (rat anti-B5R)	Dr. Don Diamond, (71)	N/A
Rabbit anti-vaccinia virus	Bio-Rad	Cat# 9503-2057
6x-His Tag Monoclonal Antibody (HIS.H8)	Invitrogen	Cat# 14-6657-82
Mouse anti-Human CD45-PE	ThermoFisher Scientific	Cat# MHCD4504-4
Goat Anti-Mouse IgG (H+L)-HRP	Bio-Rad	Cat# 1706516
Rabbit anti-Mouse IgG1 (γ-chain specific)-Peroxidase	Sigma-Aldrich	Cat# SAB3701171
Goat Anti-Mouse IgG2a heavy chain-HRP	Abcam	Cat# ab97245
Goat anti-Rat IgG (H+L)-HRP	ThermoFisher Scientific	Cat# 31470
Goat anti-mouse IgG-Alexa Fluor-647	BioLegend	Cat# 405322
Goat anti-Human IgG (H+L)-Alexa Fluor-647	ThermoFisher Scientific	Cat# A-21445
Goat Anti-Rhesus IgG(H+L)-HRP	Southern Biotech	Cat# 6200-05
Mouse Anti-rhesus IgA	Nonhuman Primate Reagent Resource	Cat# PR-0120, RRID: AB_2819303
Bacterial and virus strains		
GS1783 *E. coli* harboring MVA-BAC-TK	Dr. Don Diamond, (7)	N/A
Fowlpox virus	Dr. Bernard Moss, (8)	N/A
MVA-EBV-5-2 virus	This paper	N/A
Akata-EBV-eGFP virus	Produced from AGS-Akata-EBV-eGFP cells provided by Dr. Lindsey Hutt-Fletcher	N/A

	and Dr. Rona Scott	
Biological samples		
Serum samples from BALB/c mice	This paper	N/A
Serum samples from rhesus macaques	This paper	N/A
Saliva from rhesus macaques	This paper	N/A
PBMCs from humanized mice	This paper	N/A
Spleen from humanized mice	This paper	N/A
Kidney from humanized mice	This paper	N/A
Chemicals, peptides, and recombinant proteins		
LB broth, Miller	ThermoFisher Scientific	Cat# BP1426-500
LB agar, Miller (Powder)	ThermoFisher Scientific	Cat# BP1425-500
Chloramphenicol	Sigma-Aldrich	Cat# C0378
Kanamycin sulfate	ThermoFisher Scientific	Cat# BP906-5
L-(+)-Arabinose	Sigma-Aldrich	Cat# A3256-25G
High Fidelity Phusion Polymerase	New England Biolabs	Cat# M0531L
Agarose	Laguna Scientific	Cat# LG5002131301
Ethidium Bromide	Sigma-Aldrich	Cat# E1510
DMEM	Corning	Cat# 10-013-CM
RPMI	Corning	Cat# SH30027.01
DMEM/F-12	Corning	Cat# 10-092-CV
VP-SFM	Gibco	Cat# 11681
Fetal bovine serum	Genesee Scientific	Cat# 25-550
L-Glutamine	ThermoFisher Scientific	Cat# 25030081
CTS™ GlutaMAX™-I (100X)	Gibco	Cat# A12860
Penicillin-streptomycin	ThermoFisher Scientific	Cat# 15140122
Sodium pyruvate	Corning	Cat# 25-000-CI
HEPES	Lonza	Cat# BW17-737E
2-mercaptoethanol	Gibco	Cat# 21985023
HAT supplement (50X)	Gibco	Cat# 21060017
G418 sulfate	ThermoFisher Scientific	Cat# 10131035
12-O-tetradecanoylphorbol-13-acetate	Sigma Aldrich	Cat# 524400
Sodium butyrate	Frontier Scientific	Cat# FT-FSIJK479725
Opti-MEM	ThermoFisher Scientific	Cat# 31985062
Trypsin-EDTA, (0.25%)	ThermoFisher Scientific	Cat# 25200056
PEI MAX (Polyethyleneimine Hydrochloride)	Polysciences Inc.	Cat# 24765
Mammalian Cell Lysis Buffer	GoldBio	GB-180-100
Pierce protease inhibitor mini tablets	ThermoFisher Scientific	Cat# A32953
Laemmli SDS sample buffer, reducing (6x)	ThermoFisher Scientific	Cat# J61337.AC

Novex Tris-Glycine Native Sample Buffer (2X)	ThermoFisher Scientific	Cat# LC2673
PageRuler Plus Prestained Protein Ladder, 10 to 250 kDa	ThermoFisher Scientific	Cat# 26619
NativeMark Unstained Protein Standard	ThermoFisher Scientific	Cat# LC0725
Bovine Serum Albumin	Sigma-Aldrich	Cat# A9647
Coomassie Brilliant Blue R-250 Staining Solution	Bio-Rad	Cat # 1610436
SuperSignal West Pico PLUS Chemiluminescent Substrate	ThermoFisher Scientific	Cat# 34578
Paraformaldehyde 16% Aqueous Sol. EM grade	Electron Microscopy Sciences	Cat# 15710
VECTASTAIN® ABC-HRP Kit, Peroxidase (Rabbit IgG)	Vector Laboratories	Cat# PK-4001
DAB Substrate Kit, Peroxidase (HRP)	Vector Laboratories	Cat# SK-4100
EBV gp350/220 ectodomain (a.a.48–63)	Immune Technology Corp.	Cat# IT-005-035p
EBV gB (a.a. 23–683)	Dr. Andrew McGuire	N/A
EBV gHgL (gH: 19–679; gL: a.a. 24–137)	Dr. Andrew McGuire	N/A
EBV gp42 (a.a. 33–223)	Dr. Andrew McGuire, (5)	N/A
EBV gp42gHgL (gH: a.a. 1–679; gL: a.a. 1–137; gp42: a.a. 34–223)	Dr. Jeffrey Cohen, (9)	N/A
rhLCV gp350 (a.a. 1–739)	This paper	N/A
ABTS 2-Component Microwell Peroxidase Substrate	LGC SeraCare	Cat# 5120-0033
ABTS Peroxidase Stop Solution	LGC SeraCare	Cat# 5150-0017
Red blood cell lysis buffer	Sigma-Aldrich	
Critical commercial assays		
QIAprep Spin Miniprep Kit	QIAGEN	Cat# 27104
PureLink HiPure Plasmid Filter Maxiprep Kit	ThermoFisher Scientific	Cat# K210016
EasySep Human CD34 Positive Selection Kit II	STEMCELL	Cat# 17856
DNeasy Blood & Tissue Kit	QIAGEN	Cat# 69504
TaqMan Copy Number Reference Assay, human, RNase P	ThermoFisher Scientific	Cat# 4403326
QuantiTect Probe PCR Kit	QIAGEN	Cat# 204343
Experimental models: Cell lines		
BHK-21	ATCC	Cat# CCL-10
P3X63Ag8.653	ATCC	Cat# CRL-1580
HEK-293	ATCC	Cat# CRL-1573
Raji	ATCC	Cat# CCL-86
AGS-Akata-EBV-eGFP	Dr. Lindsey Hutt-Fletcher and Dr. Rona Scott	N/A
Experimental models: Organisms/strains		
Mouse: BALB/c	Charles River Laboratories	Strain# 028
Primate: *Macaca mulatta*	Oregon National Primate Research Center	N/A
Mouse: NOD.$Cg\text{-}Prkdc^{scid}Il2rg^{tm1Wjl}$/SzJ	The Jackson	Strain# 005557

	Laboratory	RRID: IMSR_JAX:005557
Oligonucleotides		
En Passant 64L/65L Forward primer<br>5'-<br>AATTGTACTTTGTAATATAATGATATATATTTTC<br>ACTTTATCTCATTTGATTTTTATAAAAATTGAAA<br>ATAAATACAAAGGTTC-3'	(10)	N/A
En Passant 64L/65L Reverse primer<br>5'-<br>ATTCCGAAATCTGTACATCATGCAGTGGTTAAA<br>CAAAAACATTTTTATTCCTAGTATAAAAAGGCG<br>CGCC-3'	(10)	N/A
En Passant 69R/70L Forward primer<br>5'-<br>GGAAAATTTTTCATCTCTAAAAAAAGATGTGGT<br>CATTAGAGTTTGATTTTTATAAAAATTGAAAAT<br>AAATACAAAGGTTC-3'	(10)	N/A
En Passant 69R/70L Reverse primer<br>5'-<br>ATATGAATATGATTTCAGATACTATATTTGTTCC<br>TGTAGATAATAACTAAAAATTTTTATCTAGTAT<br>AAAAAGGCGCGTAAGCGGCCGAAGCTTTCA-3'	(10)	N/A
En Passant Del3 Forward primer<br>5'-<br>TTGGGGAAATATGAACCTGACATGATTAAGATT<br>GCTCTTTCGGTGGCTGGTAAAAAATTGAAAATA<br>AATACAAAGGTTC-3'	(10)	N/A
En Passant Del3 Reverse primer<br>5'-<br>ACAAAATTATGTATTTTGTTCTATCAACTACCTA<br>TAAAACTTTCCAAATACTAGTATAAAAAGGCGC<br>GCC-3'	(10)	N/A
En Passant 44L/45L Forward primer<br>5'-<br>GAATATGACTAAACCGATGACCATTTAAAAACC<br>CCTCTCTAGCTTTCACTAAAAATTGAAAATAAA<br>TACAAAGGTTC-3'	(10)	N/A
En Passant 44L/45L Reverse primer<br>5'-<br>ATAATGTTTTTATATTATACATGTTCTAAAAGAA<br>TAATCGATACAGTTTACTAGTATAAAAAGGCGC<br>GCC-3'	(10)	N/A
En Passant 148R/149L Forward primer<br>5'-<br>ATTGATAATATAAATATGAGCATTAGTATTTCT<br>GTGGATTAATAGATTTTTATAAAAATTGAAAAT<br>AAATACAAAGGTTC-3'	(10)	N/A
En Passant 148R/149L Reverse primer<br>5'-<br>TTATGAGGTATTTAGAGATTAGAGATGATTAAT	(10)	N/A

GATCCCCATACTAGAAATTTTTATCTAGTATAA AAAGGCGCGCC-3’		
gp350 ectodomain 6x tag Reverse primer 5’- GCGGCCCTAGTATAAAAAGGCGCGCCTCATTAG TGATGGTGATGGTGATGGTGGTGCTCGAGCAGT ACTAGCATGGAGAGGTTTGA-3’	This paper	
64L/65L Forward primer 5’- TGGAATGCGTTCCTTGTGC-3’	This paper	N/A
64L/65L Reverse primer 5’- AATAGGATTATGGCAGATGGTGG-3’	This paper	N/A
69R/70L Forward primer 5’- ATTATCATAGACCTCTAACGGG-3’	This paper	N/A
69R/70L Reverse primer 5’- GTTTAGTCGTGTCTACAAAAGG-3’	This paper	
BALF5 FAM-labeled probe: 5’-TGTACACGCACGAGAAATGCGCC-3’	(103)	N/A
BALF5 forward primer: 5’-CGGAAGCCCTC TGGACTTC-3’	(103)	N/A
Reverse BALF5 primer: 5’- CCCTGTTTATCCGATGGAATG-3’	(103)	N/A
Recombinant DNA		
mH5-Kan-gp350-2A-gB-pCAGGS	This paper	N/A
mH5-Kan-gp42-2A-gL-2A-gH-pCAGGS	This paper	N/A
mH5-Kan-gp350-2A-gB-2A-gp42-2A-gL-2A-gH-pCAGGS	This paper	N/A
mH5-Kan-gp350-pCAGGS	This paper	N/A
mH5-Kan-gB-pCAGGS	This paper	N/A
mH5-gp42-pCAGGS	This paper	N/A
mH5-Kan-gH-pCAGGS	This paper	N/A
mH5-Kan-gL-pCAGSS	This paper	N/A
EBV-gp42-His-Avi-PTT3	Dr. Andrew McGuire, (5)	N/A
EBV-gH-His-Avi-PTT3	Dr. Andrew McGuire, (5)	N/A
EBV-gL-His-Avi-PTT3	Dr. Andrew McGuire, (5)	N/A
rhLCV-gp350-ecto-His-Avi-PTT3	This paper	N/A
BALF5 gBlock: ATGTCTGGGGGACTCTTCTATAACCCTTTCCTAA GACCTAATAAAGGCCTTCTGAAAAAGCCTGACA AGGAGTACCTGCGTCTCATTCCCAAGTGTTTCC AGACACCAGGCGCCGCAGGGGTGGTGGATGTG CGGGGGCCTCAGCCCCCCCTGTGCTTCTACCAA GACTCCCTGACGGTGGTGGGGGGTGACGAGGA TGGAAAGGGCATGTGGTGGCGCCAGCGTGCCC AAGAGGGCACGGCAAGGCCGGAGGCAGACACC CACGGAAGCCCTCTGGACTTCCATGTCTACGAC ATACTCGAGACGGTGTACACGCACGAGAAATG CGCCGTCATTCCATCGGATAAACAGGGGTATGT GGTGCCATGTGGCATCGTCATCAAGCTACTGGG	This paper	N/A

CCGGCGCAAGGCCGATGGGGCCAGCGTGTGTGT GAACGTGTTTGGGCAGCAGGCCTACTTCTACGC CAGCGCGCCTCAGGGTCTGGACGTGGAGTTTGC AGTCCTCAGCGCCCTCAAGGCCAGCACCTTCGA CCGCAGGACCCCCTGCCGGGTCTCGGTGGAGAA GGTCACGCGCCGTTCCATTATGGGCTACGGCAA CCATGCCGGC		
Software and algorithms		
FlowJo	BD	Version 10.7.1
GraphPad Prism	GraphPad Software	Version 9.5.1
WINPEPI	(11)	Version 11.65
Quant Studio Design & Analysis Desktop Software	ThermoFisher Scientific	Version 1.5.2
IC Measure	The Imaging Source	Version 3.0.0.503
Biorender	biorender.com	N/A
Color-blind friendly color palette selector tool	https://davidmathlogic.com/colorblind	N/A
Color-blindness simulator	https://www.color-blindness.com/coblis-color-blindness-simulator/	N/A
Other		
Bolt 4 to 12%, Bis-Tris, 1.0 mm, Mini Protein Gels	ThermoFisher Scientific	Cat# NW04122BOX
Novex WedgeWell 4 to 12%, Tris-Glycine, 1.0 mm, Mini Protein Gels	ThermoFisher Scientific	Cat# XP04122BOX
iBlot 2 system	ThermoFisher Scientific	Cat# IB21001
Nitrocellulose Membranes, 0.45 µm	Thermo Scientific	Cat# 88104
PXi Multi-Application Gel Imaging System	Syngene	N/A
BD Accuri C6	BD	N/A
NovoCyte Quanteon 4025	Agilent	N/A
LSRFortessa	BD	N/A
Salivette Cotton Swab	SciMart	Cat# SAR-511534
SimpliAmp Thermal Cycler	ThermoFisher Scientific	Cat# A24811
Sonicator	Qsonica	Cat# Q500
Optima XE-90 Ultracentrifuge	Beckman Coulter	Cat# A94471
96-well Nunc MaxiSorp flat-bottom microplates	ThermoFisher Scientific	Cat# 44240421
Filter Max F3 microplate reader	Molecular Devices	Cat# F3
QuantStudio 3 Real-Time PCR System	ThermoFisher Scientific	Cat# A28137
EVOS™ FL Digital Inverted Fluorescence Microscope	Invitrogen	Cat# AMF4300

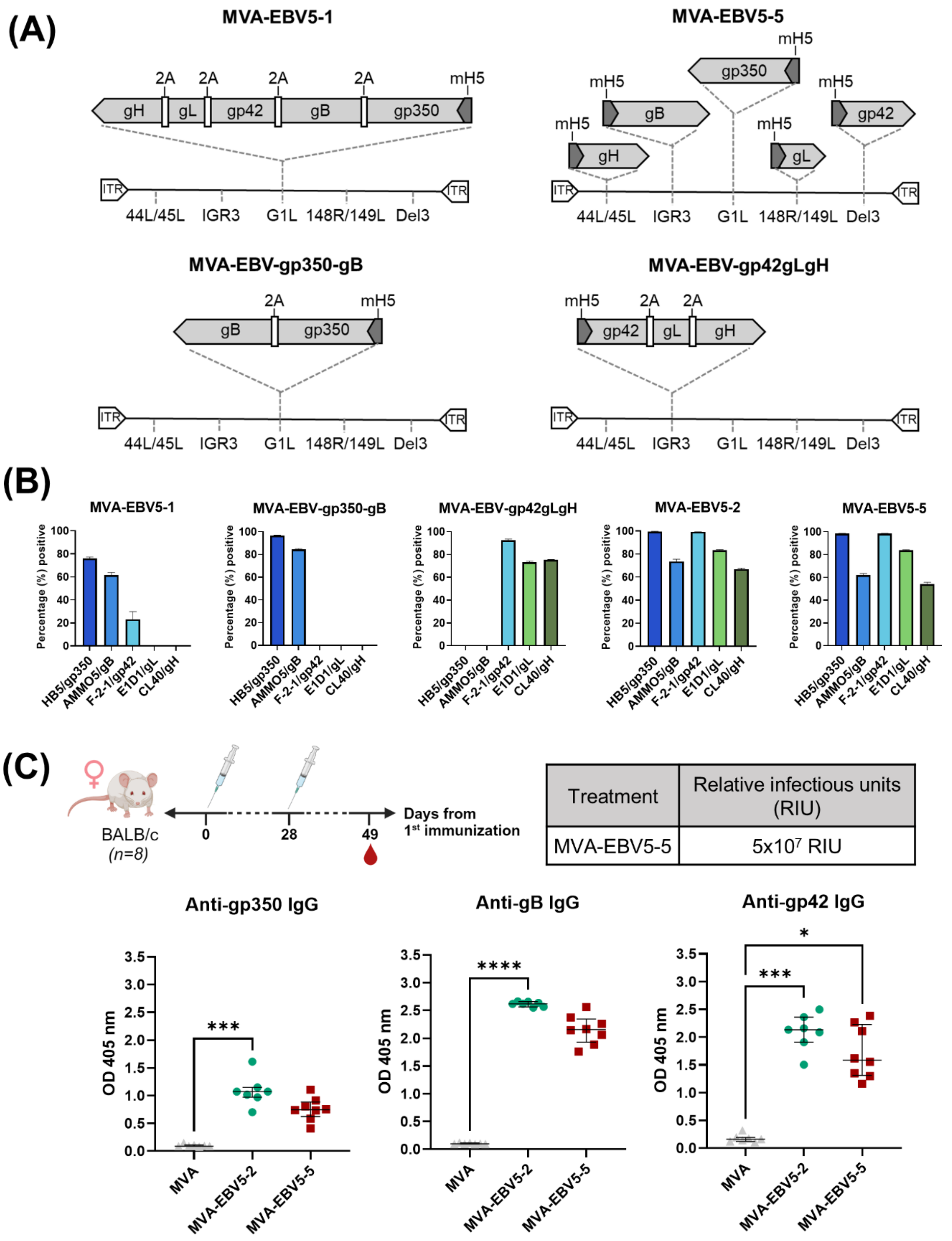

Supplementary Figure 1 (Figure S1). Preliminary characterization of recombinant MVA vectors incorporating EBV glycoproteins. (A) Schematic representations of MVA-EBV5-1, MVA-EBV5-5, MVA-EBV-gp350-gB, and MVA-EBV-gp42gLgH with EBV glycoprotein expression cassettes incorporated in the indicated MVA genomic sites. **(B)** Surface binding of the anti-gp350 monoclonal antibody (mAb) HB5, anti-gB mAb AMMO5, anti-gp42 mAb F-2-1, anti-gL mAb E1D1, and anti-gH

mAb CL40 to BHK-21 cells infected with the indicated MVA vectors was measured using flow cytometry. Each bar represents the mean percent (%) + SD of infected cells from duplicate or triplicate infections with positive signal for binding. **(C)** Female BALB/c mice were immunized with MVA-EBV5-5 ($5x10^7$ relative infectious units) on Day 0 and Day 28 (n=8), and blood was collected on Day 49. IgG binding levels to gp350, gB, and gp42 were measured using ELISA in individual mouse serum samples (1/900 dilution, bottom row), as compared to individual mouse serum samples from female BALB/c mice immunized with MVA and MVA-EBV5-2 presented in Figure 2. Each dot represents the mean of duplicate measurements for each individual animal, with the median and interquartile range shown for each group. Statistical differences were determined using Kruskal-Wallis test (* = p<0.05, *** = p<0.001, **** = p<0.0001).

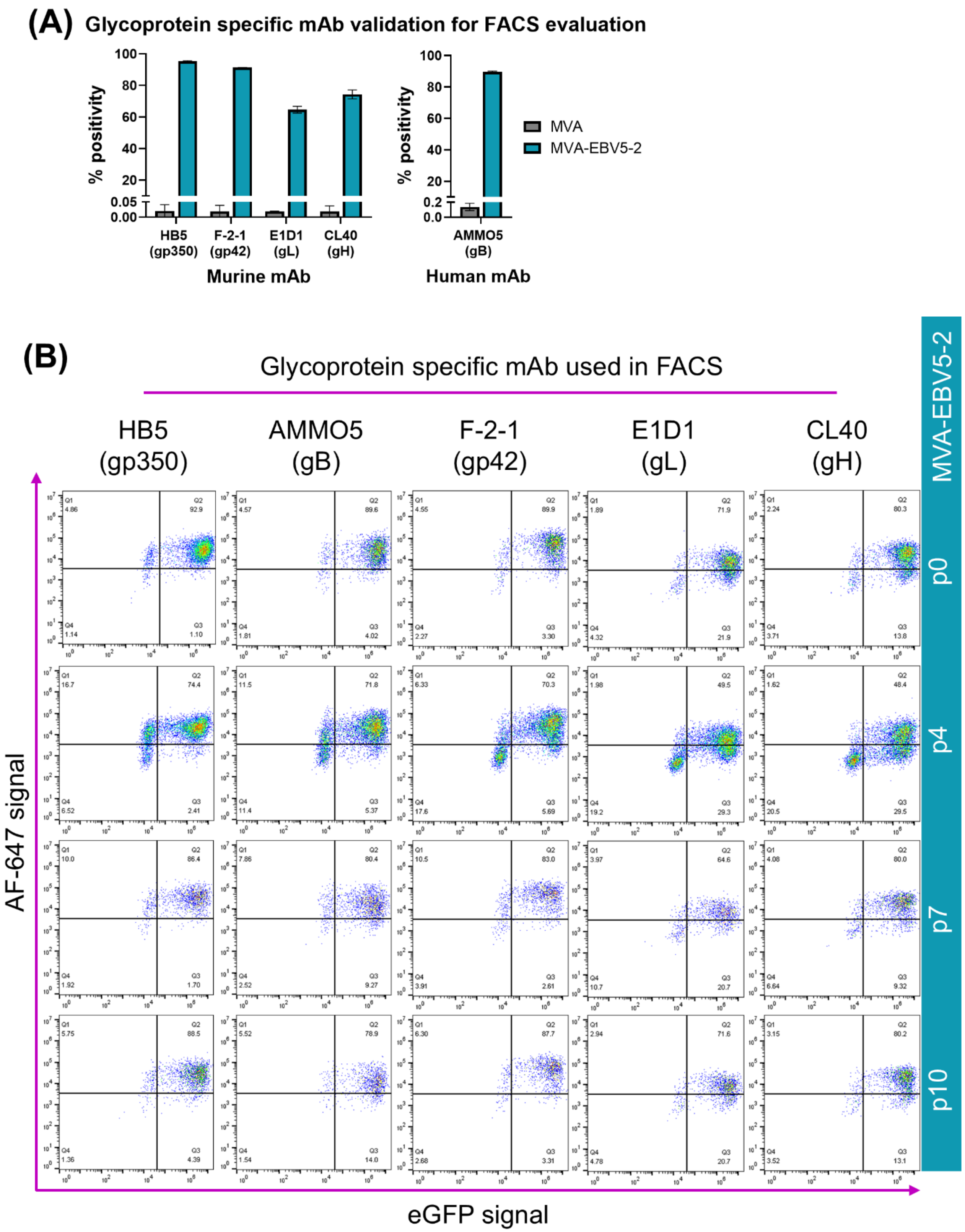

Supplementary Figure 2 (Figure S2). Validation of glycoprotein expression by recombinant MVA-EBV5-2 construct. (A) Validation of the surface binding of the indicated glycoprotein-specific monoclonal antibodies (mAbs) used for flow cytometry evaluation of MVA-EBV5-2. BHK-21 cells

infected with empty MVA virus or BHK-21 cells infected with MVA-EBV5-2 virus (p0) were incubated with anti-gp350 mAb HB5, anti-gp42 mAb F-2-1, anti-gL mAb E1D1, anti-gH mAb CL40, and anti-gB mAb AMMO5. The antigen-antibody interaction was detected using a secondary antibody conjugated to Alexa Fluor 647 (AF-647) and measured using flow cytometry. Each bar represents the mean percent (%) + SD of infected cells from triplicate infections with positive signal for binding. **(B)** Surface binding of the anti-gp350 mAb HB5, anti-gB mAb AMMO5, anti-gp42 mAb F-2-1, anti-gL mAb E1D1, and anti-gH mAb CL40 to BHK-21 cells infected with serially passaged MVA-EBV5-2 virus (p0, p4, p7, and p10) was measured using flow cytometry. Shown are representative quadrant plots from triplicate samples, with eGFP signal shown on the X-axis, a marker for MVA infection, and AF-647 signal shown on the Y-axis, corresponding to antibody staining.

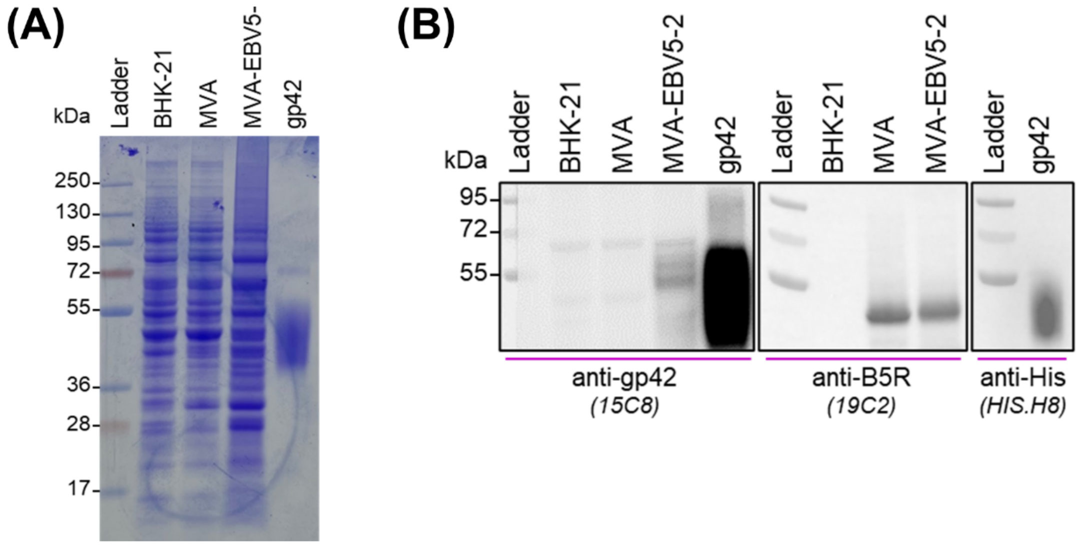

(C)

Chain	Complementary-determining region (CDR) amino acid sequence		
	CDR1	CDR2	CDR3
Heavy	SYWMQ	AIYPGDGDTRYTQKFKG	ERTPATLYAMDY
Light	KSSQSLLNSRDRKNYLT	WASTRES	KQFYNLPT

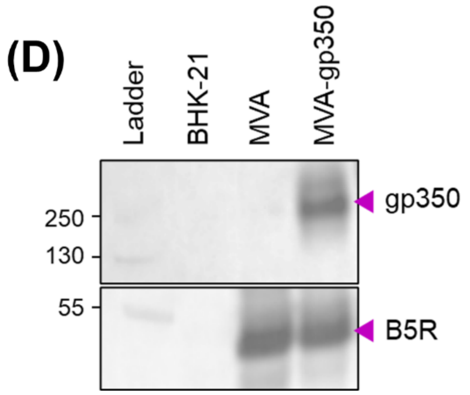

Supplementary Figure 3 (Figure S3). Validation of gp42-specific antibody 15C8, and gp350 expression by recombinant MVA-gp350 construct. (A) SDS-PAGE/Coomassie stain analysis of purified gp42 protein and total protein from uninfected BHK-21 cells, BHK-21 cells infected with empty MVA, or BHK-21 cells infected MVA-EBV5-2 used in immunoblot in panel (B). **(B)** Binding of anti-gp42 antibody 15C8 and anti-B5R hybridoma supernatant to total protein from uninfected BHK-21 cells, BHK-21 cells infected with empty MVA, or BHK-21 cells infected MVA-EBV5-2, or binding of anti-gp42 antibody 15C8 and anti-His antibody to purified gp42 protein, were assessed using immunoblot. *B5R is a vaccinia antigen and is used as a loading control.* **(C)** Complementary-determining region (CDR) amino acid sequences of the anti-gp42 antibody 15C8 heavy and light chains. **(D)** Binding of anti-gp350 antibody HB5 and anti-B5R 19C2 hybridoma supernatant to total protein from uninfected BHK-21 cells, BHK-21 cells infected with empty MVA, or BHK-21 cells infected MVA-gp350 was assessed using immunoblot. Bands corresponding to gp350 and vaccinia B5R are indicated with arrows. *B5R is a vaccinia antigen and is used as a loading control.*

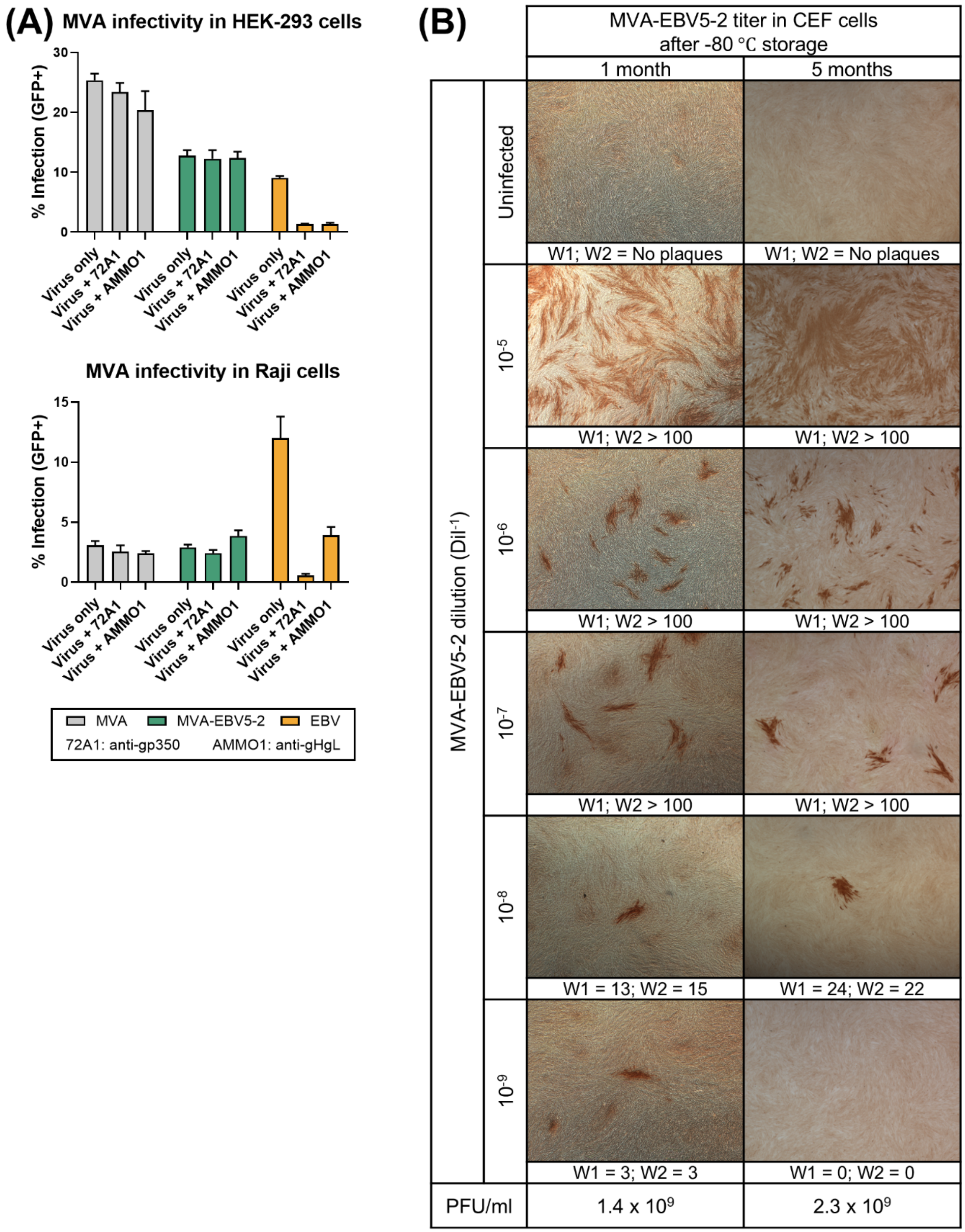

Supplementary Figure 4 (Figure S4). Evaluation of MVA-EBV5-2 tropism and storage stability. (A) The ability of MVA-EBV5-2 to infect HEK-293 cells and Raji cells, as compared to MVA wildtype (MVA) and EBV, was measured in the presence or absence of EBV-specific neutralizing antibodies 72A1 and AMMO1. Bar graphs represent the mean % infectivity + SD of triplicate measurements for each sample. **(B)** The MVA-EBV5-2 titer was measured in CEF cells after 1- or 5-month storage

periods at -80°C following virus production, via plaque-forming unit (PFU) immunostaining assay. Shown are representative micrographs of duplicate infected wells for each condition after immunostaining, together with the PFU count for each duplicate well per condition (well 1, W1; well 2, W2). Titers (PFU/ml) for both 1-and 5-month storage samples were calculated using the PFU well counts at the 10^{-8} dilution.

(A)

Treatment	Animal ID	Sex	Age
MVA-EBV5-2<br>Age Average: 12.2 Y	33479	Female	15 YO
	33480	Female	15 YO
	33487	Male	12 YO
	33469	Female	10 YO
	33470	Male	9 YO
MVA-gp350<br>Age Average: 14.8 Y	33472	Male	24 YO
	33489	Female	15 YO
	33473	Female	13 YO
	33483	Female	11 YO
	33488	Male	11 YO
UV-EBV<br>Age Average: 14.6 Y	33491	Female	24 YO
	33477	Female	15 YO
	33481	Male	14 YO
	33482	Female	13 YO
	33466	Female	7 YO

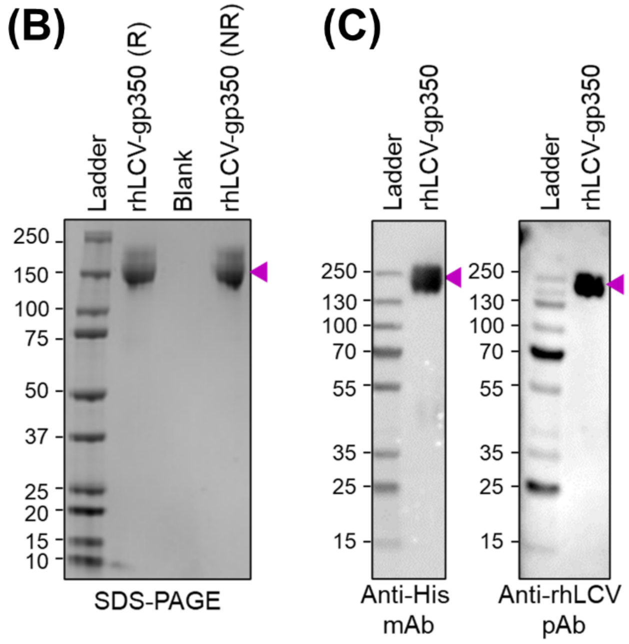

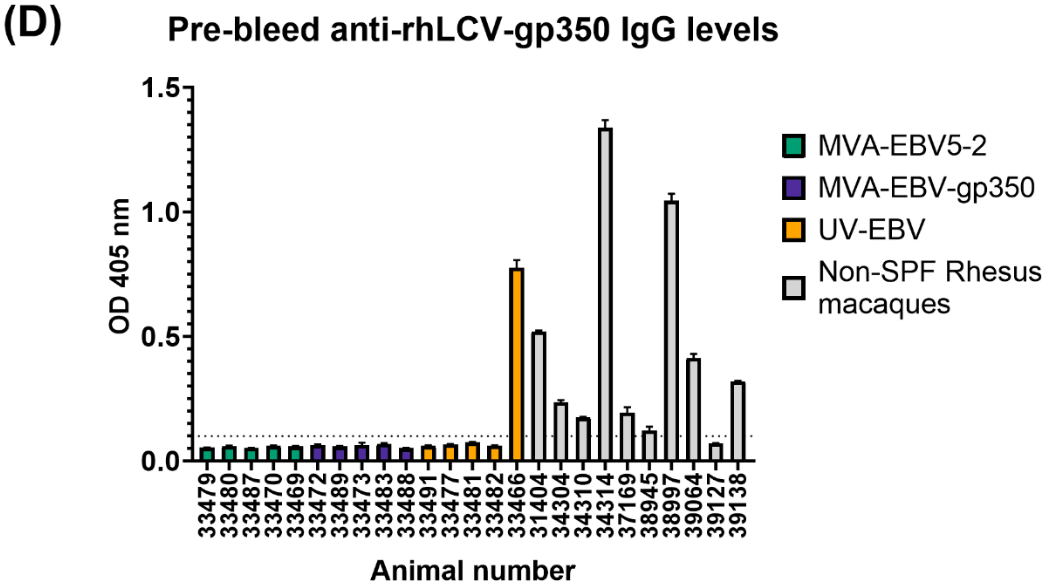

Supplementary Figure 5 (Figure S5). Experimental rhesus macaque information and rhLCV screening. (A) Table describing individual rhesus macaque demographic information and distribution into treatment groups. **(B)** SDS-PAGE/Coomassie stain analysis of purified rhLCV-gp350 used in ELISA described in panel (D). R: reducing conditions; NR: non-reducing conditions. Bands corresponding to rhLCV gp350 are indicated with arrows. **(C)** Binding of monoclonal Anti-His antibody (mAb, left) and polyclonal serum from a rhesus macaque infected with rhLCV (pAb, right) to purified rhLCV-gp350 protein used in ELISA described in panel (D) was assessed using immunoblot. Bands corresponding to rhLCV gp350 are indicated with arrows. **(D)** IgG binding to rhLCV gp350 was measured using ELISA in individual macaque serum samples prior to study start (Day -7, Figure 4) (1/100). Additional serum samples from non-SPF macaques not enrolled in the study

were also tested as controls. Each bar represents the mean of triplicate measurements for each individual animal. The dotted line represents an arbitrary OD cut-off of 0.1, picked based on the OD values distribution observed on the SPF macaque samples.

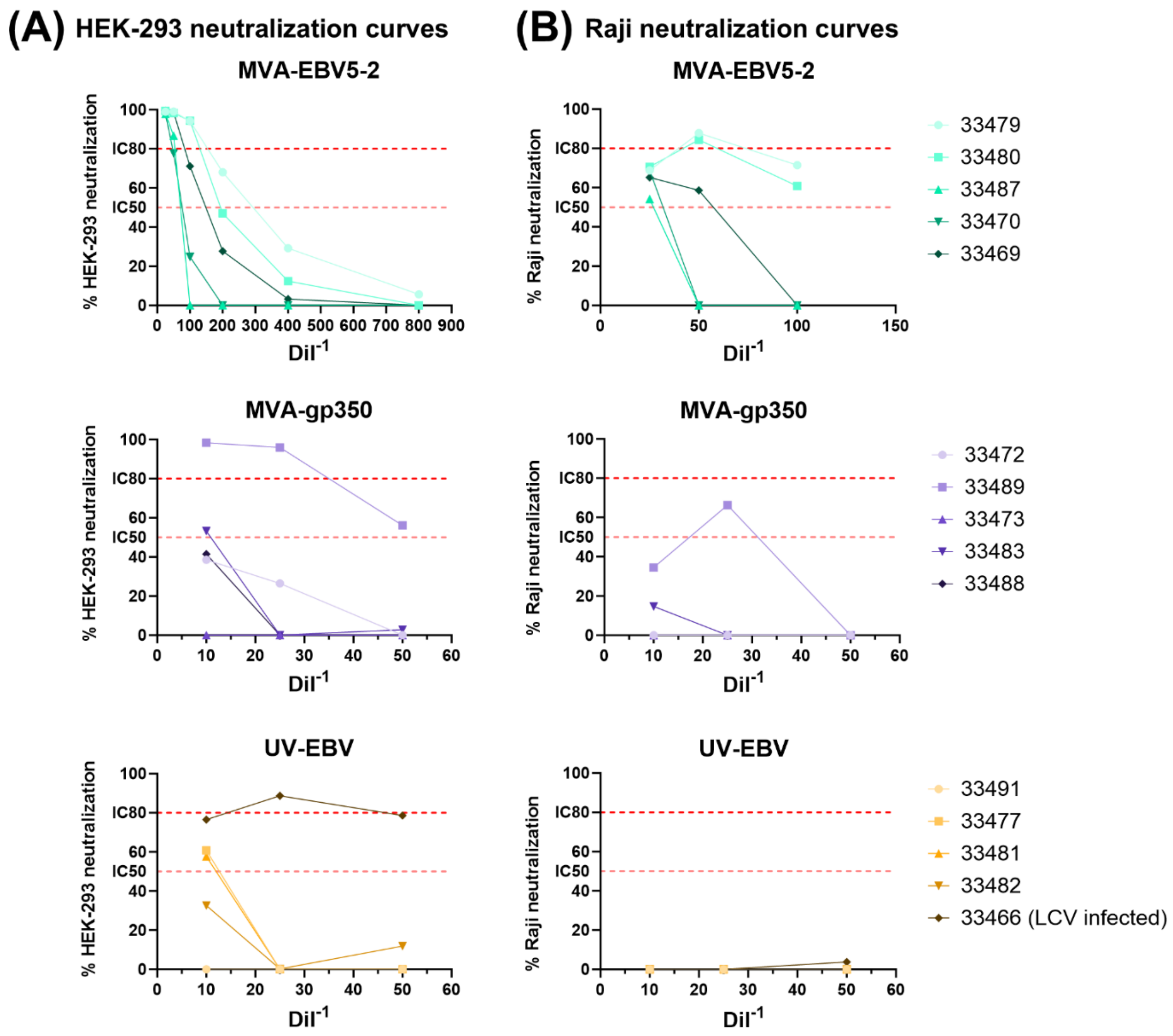

Supplementary Figure 6 (Figure S6). Neutralization titration curves for immunized rhesus macaque serum. (A-B) The ability of serially diluted Day 56 sera from individual rhesus macaques in Figure 4 to neutralize EBV infection was measured via *in vitro* neutralization assays. Individual animal titration curves for each treatment group (% neutralization) from which the Figure 5 IC50 and IC80 plots were derived are shown for HEK-293 (A) and Raji (B) cells. Samples that did not achieve neutralization as compared to Pre-immune serum were assigned a value of 0%. Top dotted line represents 80% neutralization; bottom dotted line represents 50% neutralization.

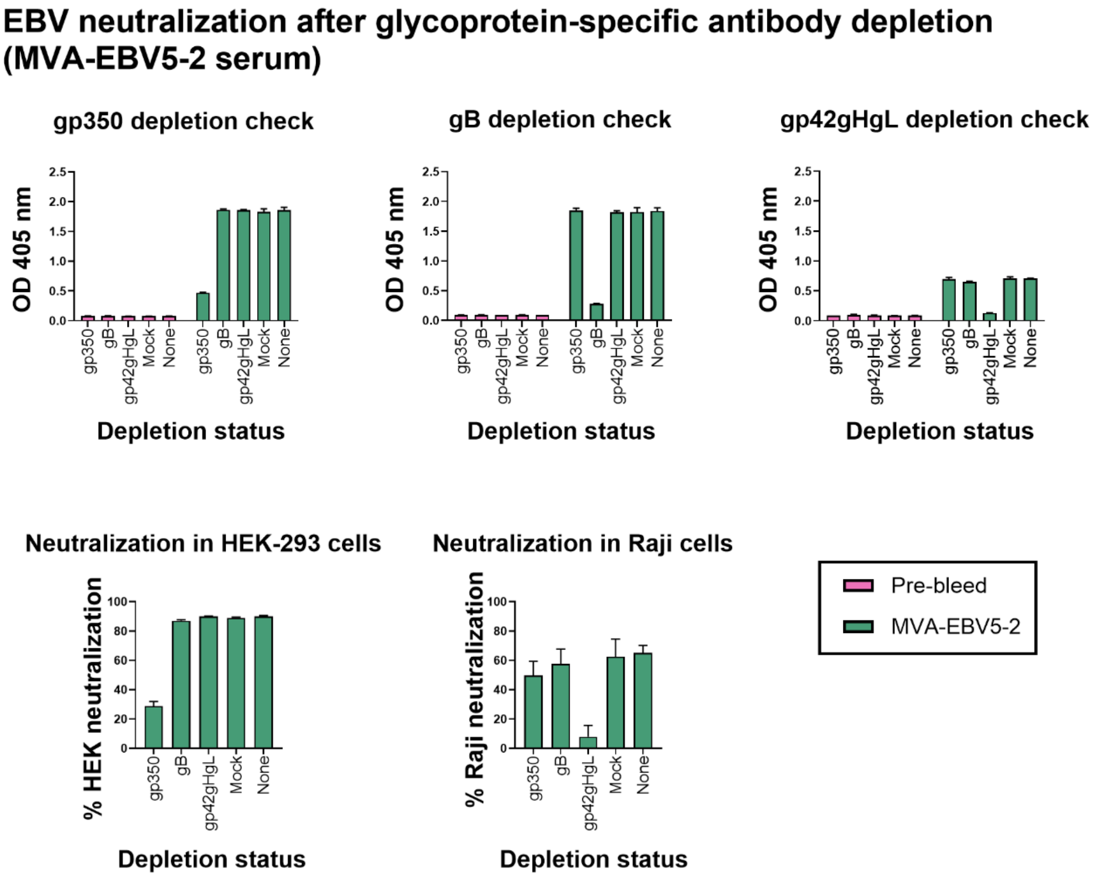

Supplementary Figure 7 (Figure S7). MVA-EBV5-2-immune rhesus macaque serum glycoprotein-specific antibody depletion. The ability of Day 56 MVA-EBV5-2 pooled sera from rhesus macaques in Figure 4 to neutralize EBV infection before and after anti-gp350, -gB, and -gp42gHgL antibody depletion was measured via *in vitro* neutralization assays. Pooled sera at a 1/12.5 dilution from each treatment group was incubated with gp350-, gB-, or gp42gHgL-coated nitrocellulose membranes, and before neutralization assays, IgG binding levels to each glycoprotein/glycoprotein complex (1/50 sera dilution) were measured using ELISA (top panel) in glycoprotein-depleted sera, sera incubated with a BSA-blocked nitrocellulose membrane (mock), or undepleted sera (none); bar graphs represent the mean + SD of duplicate measurements for each group. Glycoprotein-specific antibody-depleted sera and control sera was subsequently used in HEK-293 and Raji (bottom panel) cell neutralization assays; bar graphs represent the mean + SD neutralization of triplicate measurements for each group, at a 1/50 sera dilution.

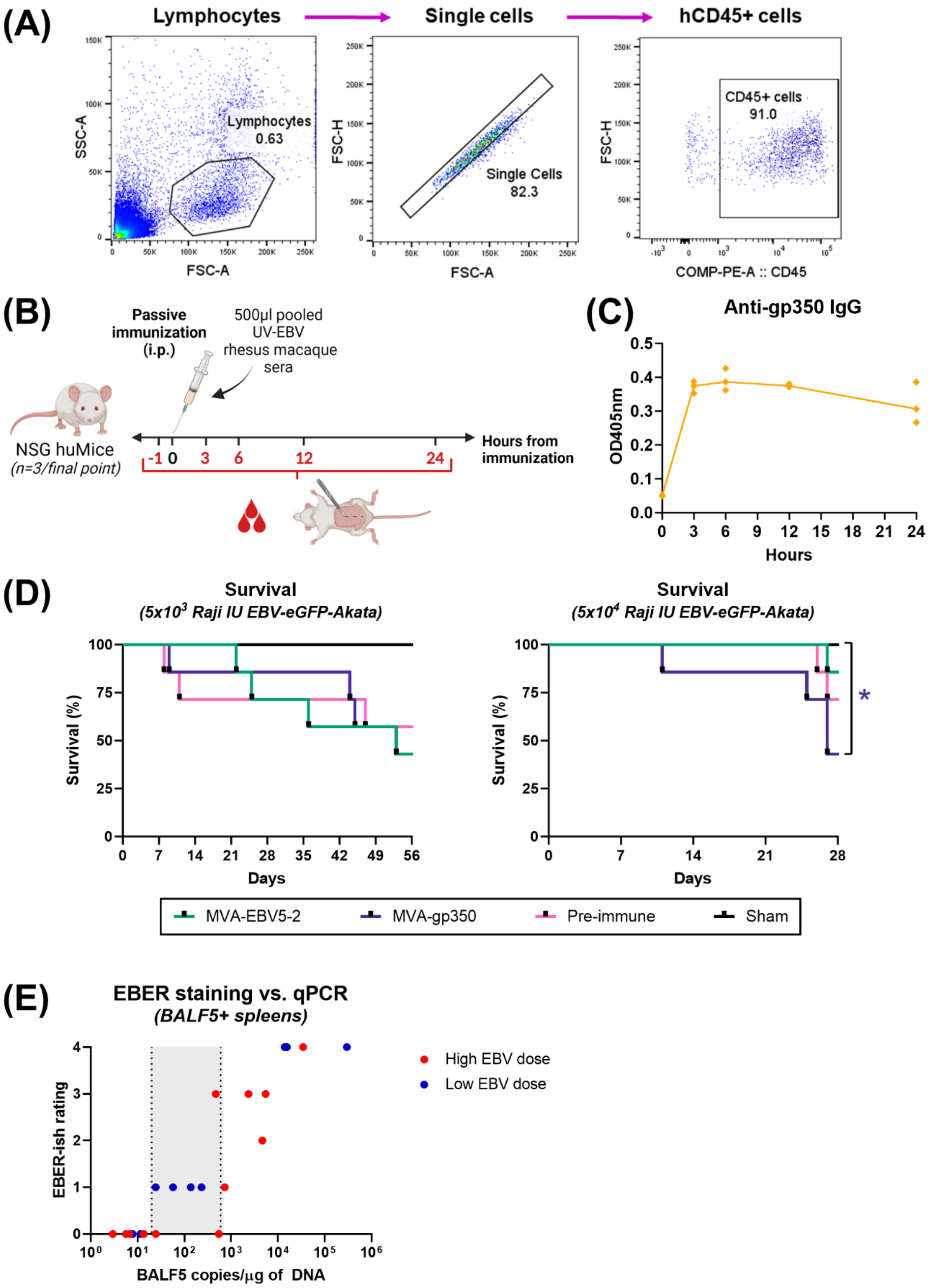

Supplementary Figure 8 (Figure S8). Assessment of human lymphocyte engraftment in NSG mice, kinetics of UV-EBV-immune rhesus macaque sera IgG in NSG huMice, and survival and comparison of spleen EBER-ish staining scores against BALF5 copies/µg in NSG huMice EBV challenge studies. (A) Percent human CD45 (hCD45)+ lymphocytes was assessed using flow

cytometry in the collected circulating blood of CD34+ cell-engrafted NSG mice from Figure 6/Table S2 and Figure 7/Table S3. Shown is the gating strategy used in the assessment. **(B)** NSG huMice were passively immunized intraperitoneally (i.p.) with 500 µl of Day 56 UV-EBV-immune sera from rhesus macaques in Figure 4 to assess the kinetics of glycoprotein-specific rhesus IgG (n=3/timepoint). Mice were sequentially euthanized at -1, 3, 6, 12, and 24 hours post-immunization for blood collection, and the intraperitoneal cavity was examined for the presence of rhesus macaque sera. **(C)** IgG binding levels to EBV gp350 were measured using ELISA in individual NSG huMouse serum samples from (B) (1/50 dilution). Each dot represents the mean of individual animal measurements in each group per timepoint, each animal was tested in triplicate. The line passes through the mean of each timepoint. **(D)** Survival curves for NSG huMice in indicated treatment groups in low (left, Figure 6) and high (right, Figure 7) EBV dose challenge studies. Statistical differences were determined using Log-rank test, but the only difference detected was between the Sham and MVA-gp350 groups in the high-dose study ($p = 0.0347$). **(E)** Comparison of EBER-ish staining scores against BALF5 copies/µg of DNA in individual mouse spleens from NSG huMice in both low (blue dots; Figure 6/Supplementary Table 2) and high (red dots; Figure 7/Supplementary Table 3) dose challenge studies.

Supplementary Table 2 (Table S2). Distribution of humanized mice into treatment groups and experimental outcomes in low EBV dose challenge study.

Treatment	Animal ID	Sex	%hCD45+ lymphocytes	Death	BALF5 copies/µg of blood DNA	BALF5 copies/µg of spleen DNA	Spleen EBER staining score
MVA-EBV5-2 sera *%hCD45+ average: 79.78*	6	F	91.35	Day 36	ND	ND	ND
	8	F	63.9	At endpoint	0.0	0.0	-
	10	F	77.4	Day 53	0.0	0.0	-
	11	F	92.55	Day 22	ND	ND	-
	17	M	85.15	At endpoint	0.0	0.0	-
	20	M	77	Day 25	0.0	0.0	-
	22	M	71.1	At endpoint	0.0	7.9	-
MVA-gp350 sera *%hCD45+ average: 79.24*	4	F	85	Day 44	1722.0	15521.7	++++
	5	F	87.35	At endpoint	0.0	0.00	-
	7	F	94.75	Day 45	0.0	11.5	-
	9	F	76.5	At endpoint	2571.3	299286.2	++++
	15	M	72.55	Day 9	ND	ND	ND
	19	M	59.3	Day 53	0.0	0.00	-
	21	M	79.2	At endpoint	0.2	137.1	+
Pre-immune sera *%hCD45+ average: 81.20*	1	M	91.3	At endpoint	120.8	14011.1	++++
	12	F	69.35	At endpoint	0.0	0.00	-
	13	F	86.75	Day 11	ND	ND	ND
	14	M	82.5	Day 8	ND	ND	ND
	16	M	72.35	Day 47	0.0	24.5	+
	24	F	91.85	At endpoint	140.2	232.9	+
	25	F	74.3	At endpoint	0.0	57.5	+
Sham *%hCD45+ average: 22.93*	3	M	21.55	At endpoint	0.0	0.0	-
	18	M	24.3	At endpoint	0.0	0.0	-

ND = Not determined

Supplementary Table 3 (Table S3). Distribution of humanized mice into treatment groups and experimental outcomes in high EBV dose challenge study.

Treatment	Animal ID	Sex	%hCD45+ lymphocytes	Death	BALF5 copies/µg of blood DNA	BALF5 copies/µg of spleen DNA	Spleen EBER staining score
MVA-EBV5-2 sera<br>*%hCD45+ average: 66.47*	7	F	63.0	At endpoint	0.0	13.6	-
	13	F	60.5	At endpoint	ND	0.0	-
	24	M	59.2	Day 27	0.0	6.8	-
	33	F	85.2	At endpoint	0.0	0.0	-
	36	M	36.3	At endpoint	66.7	2345.6	+++
	41	F	91.7	At endpoint	0.0	0.0	-
	47	F	69.4	At endpoint	0.0	0.0	-
MVA-gp350 sera<br>*%hCD45+ average: 66.40*	9	M	81.5	Day 27	880.0	5487.5	+++
	10	M	51.2	Day 11	ND	ND	ND
	11	M	64.2	Day 27	ND	3.0	-
	12	M	95.2	Day 25	ND	ND	ND
	15	F	46.9	At endpoint	20.0	735.4	+
	21	F	60.0	At endpoint	0.0	0.0	-
	44	M	65.8	At endpoint	70.0	547.0	-
Pre-immune bleed sera<br>*%hCD45+ average: 68.41*	6	F	59.4	At endpoint	20.0	4648.9	++
	25	M	61.7	At endpoint	0.0	0.0	-
	26	M	51.1	At endpoint	4026.0	34566.3	++++
	31	F	83.4	Day 26	ND	6027.7	ND
	32	F	62.9	At endpoint	0.0	468.0	+++
	40	F	70.1	At endpoint	0.0	24.7	-
	43	M	90.3	Day 27	0.0	5.6	-
Sham<br>*%hCD45+ average: 60.53*	5	F	62.0	At endpoint	0.0	0.0	-
	8	F	80.4	At endpoint	0.0	0.0	-
	19	M	57.8	At endpoint	0.0	0.0	-
	23	F	61.5	At endpoint	0.0	0.0	-
	39	F	65.8	At endpoint	0.0	0.0	-
	42	M	35.7	At endpoint	0.0	0.0	-

ND = Not determined

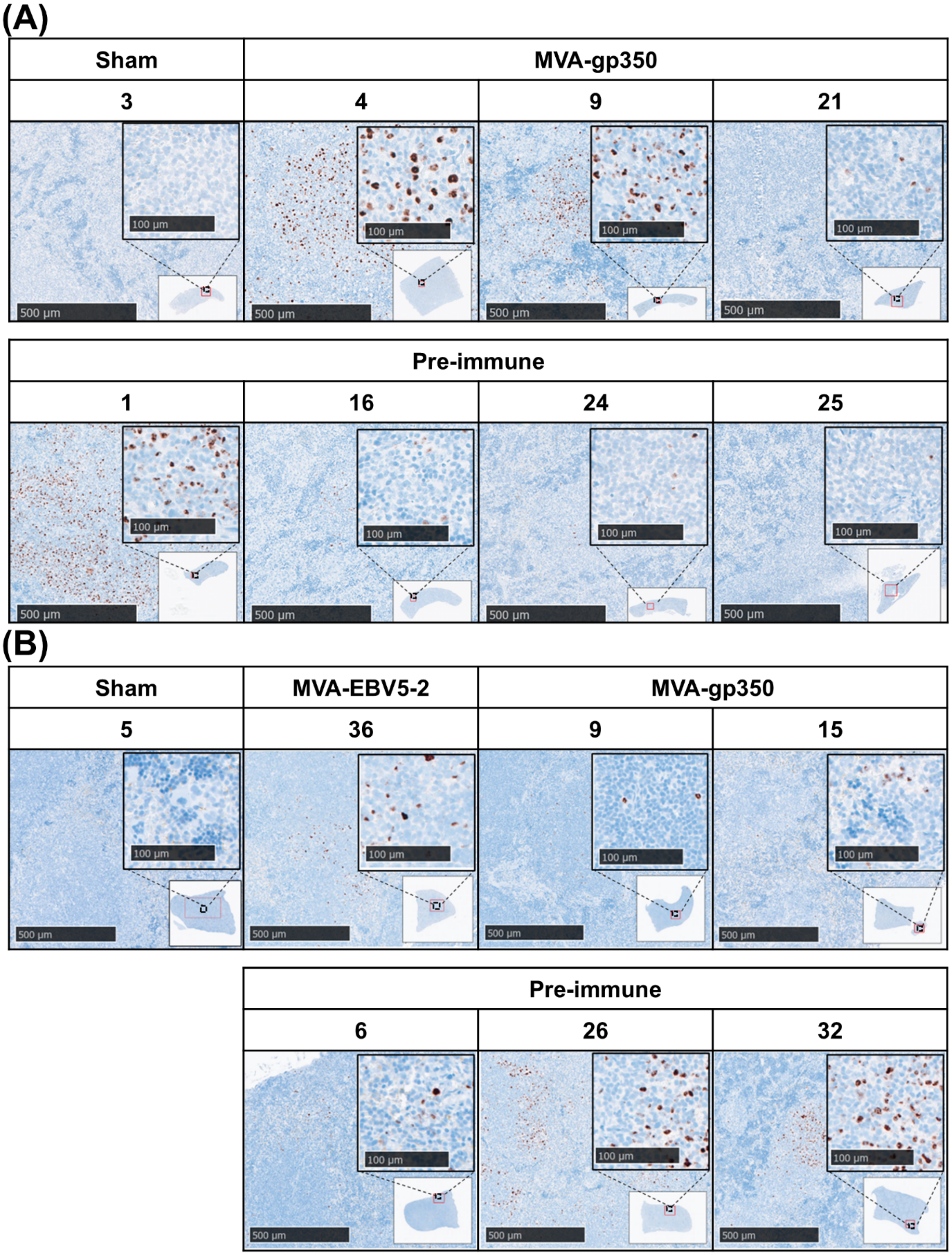

Supplementary Figure 9 (Figure S9). EBER-ish staining of spleens from NSG huMice in EBV challenge studies. (A) EBER-ish spleen staining slides for NSG huMice described in Figure 6 and Supplementary Table 2. Shown are slides for a representative control animal (Sham, #3), and all seven

EBER-positive spleens in the study with corresponding treatment group and animal # indicated. Scale: black bar denotes 500 µm in each main panel; black bar denotes 100 µm in each zoomed-in panel. EBER-ish staining appears in brown. **(B)** EBER-ish spleen staining slides for NSG huMice described in Figure 7 and Supplementary Table 3. Shown are slides for a representative control animal (Sham, #5), and all six EBER-positive spleens in the study with corresponding treatment group and animal # indicated. Scale: black bar denotes 500 µm in each main panel; black bar denotes 100 µm in each zoomed-in panel. EBER-ish staining appears in brown.

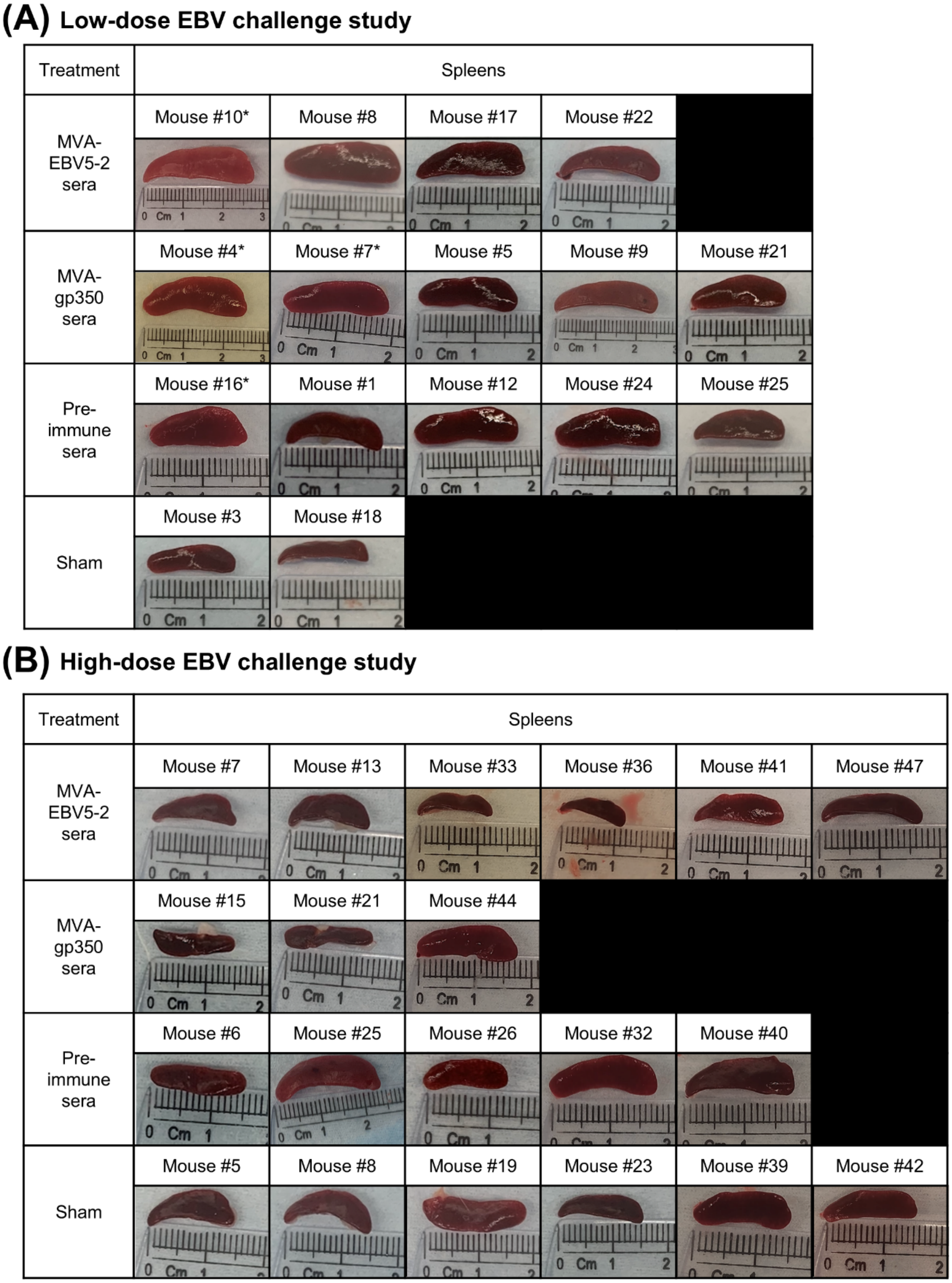

Supplementary Figure 10 (Figure 10). NSG huMice spleens from EBV challenge studies. (A-B) Images of collected spleens from NSG huMice described in Figure 6/Supplementary Table 2 (A) and in Figure 7/Supplementary Table 3 (B). Spleens marked with an asterisk in the low-dose study (A) were collected from mice that were euthanized before the experimental endpoint due to poor health. *Note that no images are available for mice in the high-dose study (B) that were euthanized early.*

Supplementary References

1. Strnad BC, Schuster T, Klein R, Hopkins RF, 3rd, Witmer T, Neubauer RH, et al. Production and characterization of monoclonal antibodies against the Epstein-Barr virus membrane antigen. Journal of virology. 1982;41(1):258-64.

2. Molesworth SJ, Lake CM, Borza CM, Turk SM, Hutt-Fletcher LM. Epstein-Barr virus gH is essential for penetration of B cells but also plays a role in attachment of virus to epithelial cells. Journal of virology. 2000;74(14):6324-32.

3. Oba DE, Hutt-Fletcher LM. Induction of antibodies to the Epstein-Barr virus glycoprotein gp85 with a synthetic peptide corresponding to a sequence in the BXLF2 open reading frame. Journal of virology. 1988;62(4):1108-14.

4. Mutsvunguma LZ, Rodriguez E, Escalante GM, Muniraju M, Williams JC, Warden C, et al. Identification of multiple potent neutralizing and non-neutralizing antibodies against Epstein-Barr virus gp350 protein with potential for clinical application and as reagents for mapping immunodominant epitopes. Virology. 2019;536:1-15.

5. Snijder J, Ortego MS, Weidle C, Stuart AB, Gray MD, McElrath MJ, et al. An Antibody Targeting the Fusion Machinery Neutralizes Dual-Tropic Infection and Defines a Site of Vulnerability on Epstein-Barr Virus. Immunity. 2018;48(4):799-811 e9.

6. Hoffman GJ, Lazarowitz SG, Hayward SD. Monoclonal antibody against a 250,000-dalton glycoprotein of Epstein-Barr virus identifies a membrane antigen and a neutralizing antigen. Proceedings of the National Academy of Sciences of the United States of America. 1980;77(5):2979-83.

7. Wussow F, Chiuppesi F, Meng Z, Martinez J, Nguyen J, Barry PA, et al. Exploiting 2A peptides to elicit potent neutralizing antibodies by a multi-subunit herpesvirus glycoprotein complex. J Virol Methods. 2018;251:30-7.

8. Domi A, Feldmann F, Basu R, McCurley N, Shifflett K, Emanuel J, et al. A Single Dose of Modified Vaccinia Ankara expressing Ebola Virus Like Particles Protects Nonhuman Primates from Lethal Ebola Virus Challenge. Sci Rep. 2018;8(1):864.

9. Bu W, Joyce MG, Nguyen H, Banh DV, Aguilar F, Tariq Z, et al. Immunization with Components of the Viral Fusion Apparatus Elicits Antibodies That Neutralize Epstein-Barr Virus in B Cells and Epithelial Cells. Immunity. 2019;50(5):1305-16 e6.

10. Chiuppesi F, Nguyen J, Park S, Contreras H, Kha M, Meng Z, et al. Multiantigenic Modified Vaccinia Virus Ankara Vaccine Vectors To Elicit Potent Humoral and Cellular Immune Reponses against Human Cytomegalovirus in Mice. J Virol. 2018;92(19).

11. Abramson JH. WINPEPI updated: computer programs for epidemiologists, and their teaching potential. Epidemiologic Perspectives & Innovations. 2011;8(1):1.
